# Identification and characterization of the *Populus trichocarpa* CLE family

**DOI:** 10.1186/s12864-016-2504-x

**Published:** 2016-03-02

**Authors:** Huibin Han, Guohua Zhang, Mengyao Wu, Guodong Wang

**Affiliations:** Key Laboratory of Ministry of Education for Medicinal Resources and Natural Pharmaceutical Chemistry; National Engineering Laboratory for Resource Developing of Endangered Chinese Crude Drugs in Northwest of China; College of Life Sciences, Shaanxi Normal University, Xi’an, 710062 Shaanxi China

**Keywords:** CLE peptide, *Populus trichocarpa*, *Arabidopsis thaliana*, Phylogenetic analysis, Transcriptional profiling

## Abstract

**Background:**

The *CLE* (*CLAVATA3/Endosperm Surrounding Region-related*) gene family encodes small signaling peptides that are primarily involved in coordinating stem cell fate in different types of plant meristems. Their roles in vascular cambium have highlighted their potential function in wood formation. Apart from recent advances on identification and characterization of *CLE* genes, little is known about this gene family in a tree species.

**Results:**

Fifty *PtCLE* genes were identified from the *Populus trichocarpa* genome and were classified into four major groups based on sequence similarity. Analysis of the genomic organization of *PtCLE* genes indicates that genome duplication, as well as the diversity in the CLE motif, have contributed to the expansion of *CLE* gene family in poplar. A comparison with functionally characterized *Arabidopsis* CLE protein sequences showed that many PtCLE proteins are closely related to their predicted *Arabidopsis* counterparts. Particularly, PtCLE3, PtCLE12, PtCLE14 and PtCLE38 comprised an identical CLE motif to AtCLE41/TDIF, which is known as a regulator of vascular cambium homeostasis, strongly supporting the idea that similar signaling pathways exist in both species to regulate wood formation and secondary growth. Transcriptome profiling revealed that *PtCLE* genes generally were differentially expressed while some *PtCLE* genes exhibited tissue-specific expression patterns. Moreover, compared to their *Arabidopsis* counterparts, *PtCLE* genes showed either similar or distinct expression patterns, implying functional conservation in some cases and functional divergence in others.

**Conclusions:**

Our study provides a genome-wide analysis of the *CLE* gene family in poplar, and highlights the potential roles of key *PtCLE* genes in the regulation of secondary growth and wood formation. The comparative analysis revealed that functional conservation may exist between PtCLEs and their AtCLE orthologues, which was further supported by transcriptomic analysis. Transcriptional profiling provided further insights into possible functional divergence, evidenced by differential expression patterns of various *PtCLE* genes.

**Electronic supplementary material:**

The online version of this article (doi:10.1186/s12864-016-2504-x) contains supplementary material, which is available to authorized users.

## Background

Small regulatory peptides, a growing class of signaling molecules mediating cell-cell communication, are essential for plant growth, development and responses to environmental stimuli [1–6]. The CLE (CLAVATA3/Endosperm Surrounding Region-related) peptide family is one of the well-studied peptide families in plants. The *CLE* genes have been found in many plant species and some plant parasitic nematodes, while the functions of most *CLE* genes are still unknown [2, 3, 7–13]. However, accumulated data have revealed that *CLE* genes played vital roles in stem cell homeostasis of different types of plant meristems including the SAM (Shoot Apical Meristem; AtCLV3), the RAM (Root Apical Meristem; AtCLE40, AtCLE19 and AtCLE22), the vascular meristem (AtCLE41/TDIF) and the root nodule meristems (LjCLE-RS1/2; MtCLE12/13; GmRIC1/2) [14–27].

Other than their roles in stem cell homoeostasis, *CLE* genes have been found to participate in a range of biological processes [2–6]. *AtCLE1*, *AtCLE3*, *AtCLE4*, and *AtCLE7*, for example, were predominantly expressed in the *Arabidopsis* root pericycle, and their expressions were induced under nitrogen-deficient conditions [28]. Over-expression of *AtCLE1*, *AtCLE3*, *AtCLE4*, and *AtCLE7* repressed the emergence and growth of lateral roots, which required CLV1, suggesting that CLV1 mediated a nitrogen-responsive CLE peptide signaling pathway that negatively regulated later root development under nitrogen deficiency [28]. *AtCLE8* is specifically expressed in the endosperm and young embryos [29]. The mutation of *AtCLE8* caused smaller and defective seeds/embryos, while ectopic expression of the *AtCLE8* gene resulted in larger seeds/embryos, indicating that AtCLE8 played crucial roles in embryogenesis and endosperm development [29]. Overexpression of *HgCLE1*, a *CLE*-like nematode gene, resulted in a *wus*-like phenotype and a short-root phenotype. Consistently, overexpression of *HgCLE1* rescued the *clv3-1* mutant phenotype [30]. Further studies have shown that multiple receptors, including CLV1, RPK2, CRN/SOL2 and CLV2, are required for the successful nematode infection of *Arabidopsis* roots [31, 32].

It has been shown that a number of *CLE* genes, including *AtCLE6*, *AtCLE10*, *AtCLE19*, *AtCLE41*/*TDIF* and *AtCLE44*, played roles in vascular development [5, 33, 34]. In particular, exogenous application of AtCLE41/TDIF peptides inhibited xylem vessel differentiation, but had no effect on the SAM and/or RAM development. Consistently, over-expression of *AtCLE41/TDIF* resulted in a xylem vessel strand-discontinuous phenotype in a PXY/TDR-dependent manner [18, 22, 23]. Intriguingly, both over-expression and exogenous peptide application promoted cambial cell proliferation [19, 23]. In combination, the data suggested that AtCLE41/TDIF promoted the proliferation of vascular cambium cells while preventing them from differentiating into xylem through the TDR/PXY receptor [19, 22, 23]. Recently, it has been suggested that the AtCLE41/TDIF-PXY/TDR signaling module is evolutionarily conserved on regulating the secondary growth in poplar tree species [35]. By tissue-specific over-expression of *PttCLE41* and *PttPXY* genes, Etchells and colleagues (2015) generated poplar trees that exhibited enhanced growth and increased wood formation [35].

Poplar has been proposed as a model plant in understanding the molecular basis of tree growth and development, particularly the formation of wood which is commercially used for manufacturing, such as fuel and construction materials [36]. However, little is known about *CLE* genes in this economically important tree species. As the conservation of their fundamental roles in the regulation of maintenance and differentiation of meristematic tissues, particularly the cambium, as well as other cellular processes, it is of great interest to study the *CLE* gene family in poplar, with an focus on *CLE* genes exhibiting expression in vascular tissues which might be important for wood formation. With the availability of the genome sequence of poplar (*Populus trichocarpa*), we carried out a genome-wide analysis to identify *CLE* genes as a first step to gain insights into their potential roles in various aspects of poplar growth and development, enabling a better understanding of the *CLE* gene family in a tree species.

## Results and discussion

### Identification and annotation of the CLE family in *Populus trichocarpa*

Systematic TBLASTN and BLASTP analyses were performed using previously reported CLE proteins and CLE motifs from various plant species as queries searching against the *Populus trichocarpa* genome (http://www.phytozome.net/). The retrieved candidate genes were then filtered for proteins with an N-terminal signal peptide and a C-terminal conserved CLE motif [9]. The analysis was iterated until no new CLE candidate was identified. As a result, a total of 50 *PtCLE* (*Populus trichocarpa CLE*) genes were identified (Table 1). Twenty-six *PtCLE* genes were reported previously [11], thus our current work identified 24 additional PtCLE members (Table 1).

**Table 1:** A list of fifty *PtCLE* genes identified in this study

Gene symbol	Gene ID	Group	Protein length(AA)	Motif (13 AA)	Gene symbol	Gene ID(2.2)	Group	Protein length(AA)	Motif (13 AA)
***PtCLE1***	Potri.001G016100.1	I	113	EREVPTGPDPLHH	*PtCLE26*	Potri.009G029000.1	V	118	IHKSSSGPNPVGN
*PtCLE2*	Potri.001G049700.1	V	137	AHEVPSGPNPESN	***PtCLE27***	Potri.009G068800.1	I	84	KRKVYTGPNPLHN
***PtCLE3***	Potri.001G075200.1	V	162	AHEVPSGPNPISN	***PtCLE28***	Potri.010G039800.1	II	109	KRRVPNGPDPIHN
***PtCLE4***	Potri.001G217500.1	V	109	LRAAPSGPDPLHH	***PtCLE29***	Potri.010G111200.1	I	104	KRTIHTGPNPLHN
*PtCLE5*	Potri.001G237700.1	V	109	IHKSPSGPNPVGN	*PtCLE30*	Potri.010G124900.1	V	105	KRRVPSCPDPLHN
*PtCLE6*	Potri.001G274200.1	I	88	KRKIFTGPNPLHN	***PtCLE31***	Potri.010G130400.1	I	110	KRLVPTGPNPLHH
***PtCLE7***	Potri.001G376100.1	III	73	FRLSPGGPDPRHH	*PtCLE32*	Potri.010G160600.1	IV	156	KRLVPSGPNPLHN
*PtCLE8*	Potri.001G376200.1	III	79	DRLSPGGPDPQHH	*PtCLE33*	Potri.010G169300.1	II	93	KRRVRRGSDPIHN
*PtCLE9*	Potri.002G121800.1	II	79	KRKVPNASDPLHN	*PtCLE34*	Potri.010G206700.1	V	86	IHKAPSGPSPIGN
***PtCLE10***	Potri.002G226300.1	II	74	KRRVPAGPNPLHN	***PtCLE35***	Potri.011G063700.1	III	76	KRVSPGGPDAQHH
***PtCLE11***	Potri.002G228000.1	IV	87	RRKIPAGPNPLHN	***PtCLE36***	Potri.011G096800.1	III	73	FRLSPGGPDPRHH
*PtCLE12*	Potri.002G241300.1	V	131	AHEVPSGPNPISN	***PtCLE37***	Potri.011G096900.1	III	78	DRVSPGGPDPHHH
*PtCLE13*	Potri.003G124000.1	III	88	YRAVPGGPNPLHN	***PtCLE38***	Potri.012G019400.1	V	116	AHEVPSGPNPISN
*PtCLE14*	Potri.003G156000.1	V	115	AHEVPSGPNPISN	*PtCLE39*	Potri.012G059600.1	I	107	KRLVPTGPNPLHH
*PtCLE15*	Potri.003G178500.1	V	134	FHEVPSGPNPESN	*PtCLE40*	Potri.012G138100.1	III	87	HKAVPGGPNPLHN
*PtCLE16*	Potri.004G053700.1	III	66	KRVSPGGPDAKHH	*PtCLE41*	Potri.012G138200.1	IV	87	RRLVPSGPNPLHN
*PtCLE17*	Potri.005G034000.1	V	66	SRAVPSGPDPLNN	*PtCLE42*	Potri.013G023500.1	V	74	NRVVPSCPDPIHN
*PtCLE18*	Potri.006G036700.1	I	99	KRKVYTGPNPLHN	***PtCLE43***	Potri.013G119100.1	III	79	KRLSPGGPDPKHH
***PtCLE19***	Potri.008G086100.1	II	123	KRRAPRGSDPIHN	***PtCLE44***	Potri.014G156600.1	II	74	KRKVPTGSNPLHN
***PtCLE20***	Potri.008G093500.1	IV	118	KRLVPSGPNPLHN	***PtCLE45***	Potri.015G139900.1	III	88	HKLVPGGPNPLHN
***PtCLE21***	Potri.008G115600.1	I	113	KRLVPTGPNPLHH	*PtCLE46*	Potri.015G140000.1	IV	152	RRLVPCGPNPLHN
*PtCLE22*	Potri.008G120800.1	V	106	KRRVPSCPDPLHN	***PtCLE47***	Potri.017G074600.1	II	97	KRRVPNGPDPIHN
***PtCLE23***	Potri.008G130800.1	I	103	KRIIHTGPNPLHN	***PtCLE48***	Potri.019G090800.1	III	85	DRLSPGGPDPHHH
***PtCLE24***	Potri.008G191500.1	II	107	KRKVPNGPDPIHN	*PtCLE49*	Potri.019G090900.1	III	85	DRLSPEGPNHEHH
***PtCLE25***	Potri.009G020300.1	V	100	LRAVPSGPDPLHH	***PtCLE50***	Potri.019G091100.1	III	76	KRISPGGPDPKHH

A complete list of PtCLEs identified in the present study. The names in bold indicate the PtCLE proteins which were also identified in Oelkers et al. [11]

**Fig. 1:**
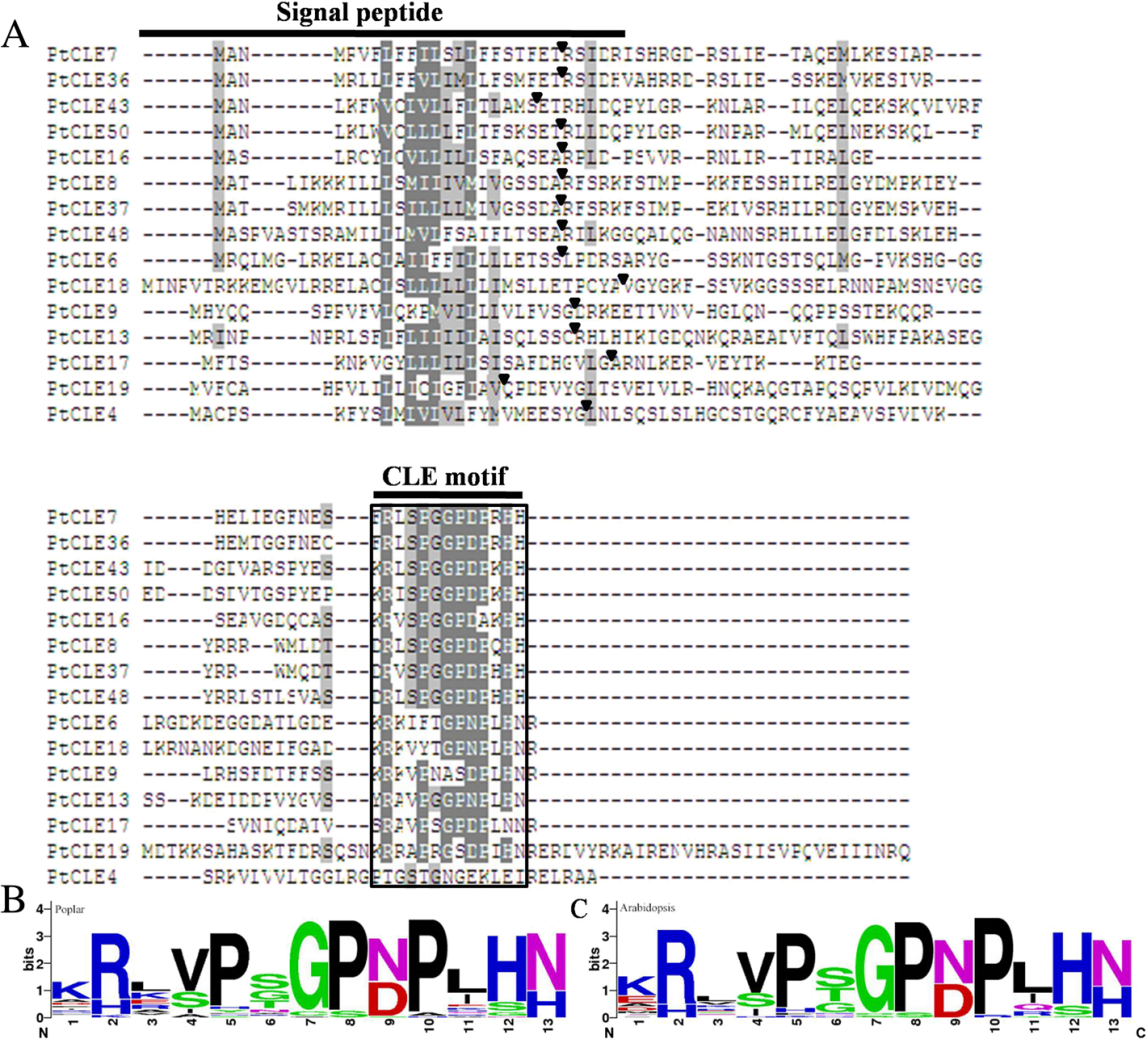
Multiple sequence alignment of representative PtCLE proteins and the consensus sequence for the CLE motifs of poplar and *Arabidopsis*. **a** The multiple sequence alignment of representative PtCLE proteins. The predicted proteolytic cleavage sites are indicated by the small arrowheads. The CLE motif is boxed. **b**-**c** Weblogo plots were used for display the CLE motifs of poplar and *Arabidopsis*

Similar to *Arabidopsis* CLE proteins, PtCLEs displayed few sequence features with each other, apart from the secretion signals and the CLE motifs (Fig. 1; Additional files 1, 2, 3, 4, 5, 6 and 7). In line with the AtCLE members, the presence and location of putative N-terminal signal peptide cleavage sites were predicted in each PtCLE (Fig. 1; Additional file 1). It has been shown that deletion of the putative CLE signal peptide inactivated the CLE protein activity in vivo, suggesting that the signal peptide is essential for in vivo functions of CLE peptides [37].

The CLE proteins contain one or more C-terminal conserved CLE motif(s), which was reported to be a 12–13 amino acid hydroxyprolinated, triarabinosylated peptide, and was the functional domain of CLE proteins [38, 39]. MEME (Multiple Expectation Maximization for Motif Elicitation) was employed to investigate the presence and distribution of CLE motifs in all PtCLE proteins. Only one single CLE motif was found to be present across all PtCLEs (Fig. 1; Table 1; Additional files 1, 2, 3, 4, 5, 6 and 7). The presence of multiple CLE domains was not observed in any of the PtCLE proteins although CLE proteins containing multiple CLE domains have been reported previously (Table 1; Fig. 1; Additional files 1, 2, 3, 4, 5, 6 and 7; [10, 11]).

The CLE motif, a segment that contains the mature CLE peptide sequence, is highly conserved across all CLE proteins [37, 40]. As expected, the consensus sequences of the CLE motif between AtCLE and PtCLE are highly conserved (Fig. 1b-c; Additional file 3; Additional file 5), suggesting functional conservation between PtCLEs and AtCLEs. Similar to AtCLEs, residues R2, P5, G7, P8, P10 and H12 of the CLE motifs in PtCLEs are highly conserved (Fig. 1b-c). Only moderate conservation was observed for amino acids (V/S)4, (N/D)9 and (N/H)13, although a similar probability of occurrence presented in both AtCLEs and PtCLEs (Fig. 1b-c; Additional file 3; Additional file 5). These conserved residues might provide a framework for the physical binding with their presumed receptors. Studies have been reported that residues D, H, G, P5, R and P10 of the CLE domain were critical for proper AtCLV3 function in SAM as evidenced by Ala-substitutions [41]. In addition, residues in the flanking sequences and the hydroxylation/arabinosylation modifications of residue P8 are also critical to the AtCLV3 function [42–44]. Furthermore, a Gly-to-Thr substitution in the CLE motifs resulted in a strong dominant-negative effect [26]. However, to what extent the conservation of these residues in the CLE motif across poplar and *Arabidopsis* could reflect their functional relevance awaits further investigation. Furthermore, the CLE motif exhibited residue divergence at positions 1, 3, 6 and 11 (Fig. 1b-c, Additional file 3; Additional file 5), which may provide the basis for distinct functions of the individual PtCLEs and/or the specificity of the putative receptor(s) binding.

Four or five residues proximal to the CLE motif at the N-terminus are required for proper endoproteolytic processing and optimal function in stem cell regulation [44, 45]. A comparison of the six residues (6-AA) proximally adjacent to the CLE motifs revealed high divergence across all PtCLEs, but a degree of residue conservation was found for multiple PtCLEs (Additional files 6 and 7). A Lys residue is presented before the conserved Arg residue of the 12-AA CLE motif in many PtCLEs (Additional files 6 and 7). This may suggest that the importance of this residue for endopeptidase recognition, which has been shown in the case of AtCLV3 and AtCLE1 [44]. Additionally, 17 out of 50 PtCLEs carried an Arg residue immediately following the CLE motif at the C-terminus, indicating a possible decrease of peptide activity as has been reported previously [46].

### The PtCLE proteins are classified into four major distinct groups

**Fig. 2:**
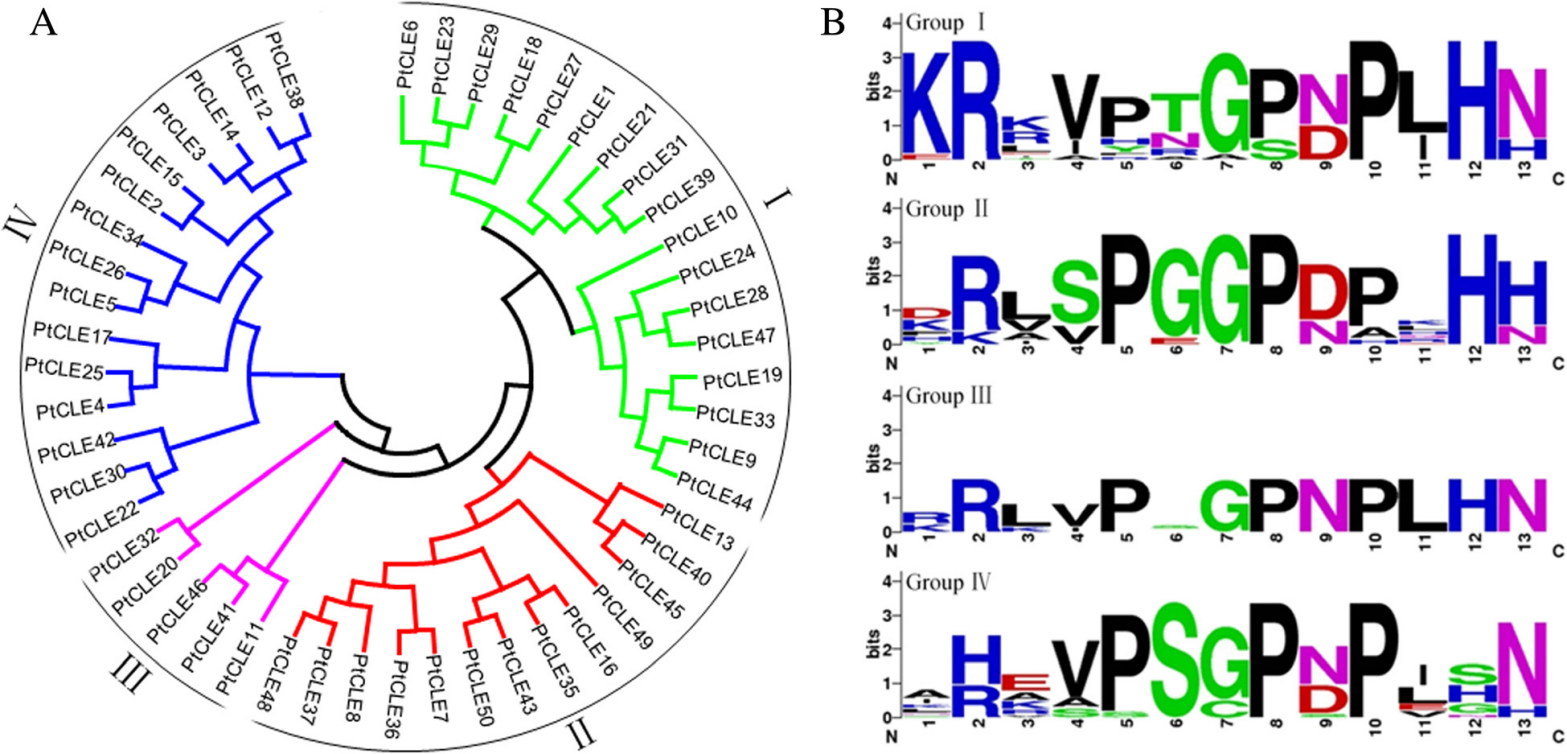
The PtCLE proteins are classified into four major groups. **a** Phylogenetic analysis of PtCLE proteins. The tree was generated from the alignment of the CLE motifs of all PtCLE protein sequences with 1000 bootstrap replicates. The distinct groups are shown by colored branches. **b** Weblogo representation for the CLE motifs for each of the four groups

Although PtCLE proteins shared little sequence similarity, the CLE motifs were well conserved. Therefore, all the CLE motif sequences, as well as the full length proteins, were used as the basis to build phylogenetic trees separately. Phylogenetic analyses using several methods supported the classification of PtCLE proteins into four major groups (Fig. 2a; Additional file 8). The CLE motifs of the four groups were aligned, which resulted in consensus sequences supporting for classification of these four groups (Fig. 2b).

The consensus sequences of the CLE motifs in all groups (positions 7–13) were highly conserved with five residues that were almost invariant, except for position 11 of Group II and position 12 of Group IV (Fig. 2b). However, residue divergence across the first six N-terminal residues of the CLE motif was observed in all groups, especially in Group IV, in which high variance was observed (Fig. 2b). The CLE motifs of Groups I, II and III lacked the conservation of the Ser residue at position 6, which was invariant in Group IV (Fig. 2b). The Lys residue at position 1 of Group I was highly conserved, whereas the residue at the same position of other groups was rather variable (Fig. 2b). Group II contained a group-specific Ser residue at position 4, which may be largely responsible for its separation into a distinct group (Fig. 2b). However, whether the conserved residues and/or distinct group-specific residues contribute to CLE functionalities requires biological validation.

Previously, CLE proteins identified from various plant species were categorized into thirteen groups [11]. A closer examination of the CLE consensus sequences revealed that Groups I, II, III and IV of PtCLEs corresponded to Groups 7, 2, 9 and 5 presented in [11], respectively. The comparison indicated a similar signature of the CLE motifs in both classifications. It was reported that *Arabidopsis* CLE was classified into four functional groups based on the effects of peptide treatment on plants [47], which was well correlated with the phylogenetic analysis of AtCLEs [23, 48, 49]. The classification presented in [11] contained at least one functional CLE in each group, which helped to understand the possible function(s) of individual PtCLE group. Nevertheless, the correlation of phylogenetic analyses between ours and [11] implied strong functional similarities between interspecies orthologs as validated by many functional characterized *CLE* genes from *Arabidopsis*, rice, *Medicago*, *Lotus japonicus* and soybean [2, 3]. For instance, Group IV members, which correspond to the Group 5 as classified in [11], were predicted to confer similar phenotypic effects on vascular development in poplar to those observed in *Arabidopsis* [11, 18, 22, 23]. However, determining whether these predicted gene functions are evolutionarily conserved requires further biological investigation.

### Genomic organization of *PtCLE* genes

**Fig. 3:**
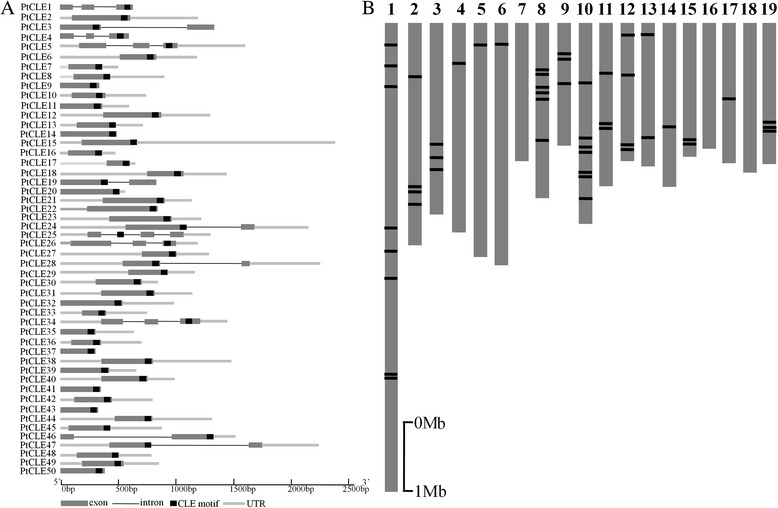
Genomic organization of *PtCLE* genes. **a** The *PtCLE* gene structure is presented using gray boxes for exons, lines for the introns, and bold lines for UTRs, respectively. The black boxes mark the CLE motifs. **b** Physical locations of *PtCLE* genes on *Populus* chromosomes. The *PtCLE* genes are located according to the JGI *Populus* v2.2 gene annotation. The scale bar represents 1.0 Mb

Similar to *AtCLE* genes, *PtCLE* genes often lacked introns. Only thirteen *PtCLE* genes contained intron(s), seven of which contained one intron and six of which contained two introns (Fig. 3a). *PtCLE* genes scattered over on different chromosomes although some clustering can be observed (Fig. 3b). Furthermore, some of the *PtCLE* genes were found to be located adjacently to each other (Table 1; Fig. 3b). For instance, *PtCLE7* and *PtCLE8*, *PtCLE36* and *PtCLE37*, *PtCLE40* and *PtCLE41*, *PtCLE45* and *PtCLE46* were located in tandem on chromosomes 1, 9 12 and 15, respectively. Additionally, *PtCLE48*, *PtCLE49* and *PtCLE50* were organized sequentially in tandem on chromosome 19 (Table 1; Fig. 3b). However, sequence comparison within those tandem pairs showed low sequence similarity, and the CLE motifs were not totally identical, implying that these genes might not arise from recent tandem duplication events (Table 1; Fig. 3b; Additional files 2 and 3). These observations may indicate, in some cases, that diversity in the CLE motifs was favored during evolution which may give rise to distinct roles of PtCLEs and expansion of the *PtCLE* gene family.

Interestingly, a number of *PtCLE* genes located on different chromosomes encoded identical, or nearly identical CLE motifs, suggesting that these *PtCLE* genes were possibly duplicated genes arising from segmental duplication events (Additional file 9). For instance, the CLE motifs of the positionally adjacent pairs PtCLE7/PtCLE8 were almost identical to that of PtCLE36/PtCLE37, while those of PtCLE40/PtCLE41 were nearly identical to that of PtCLE45/PtCLE46 (Table 1; Fig. 3; Additional files 2 and 3). Moreover, PtCLE3, 12, 14 and 38 comprised identical CLE motifs, while PtCLE21, 31 and 39 shared the same CLE motifs (Table 1; Additional file 9). A set of five pairs, PtCLE7/PtCLE36, PtCLE18/PtCLE27, PtCLE20/PtCLE32, PtCLE22/PtCLE30 and PtCLE28/PtCLE47, carried identical CLE motifs within pairs (Table 1; Additional file 9). These results suggested that genome-scale duplication of *PtCLE* genes occurred in different regions of poplar chromosomes. In tomato, neighboring *SlCLE* genes, sharing no significant similarity within pairs, were found on different chromosomes, suggesting that these neighboring *SlCLE* were not likely to arise through tandem duplication [13]. However, it was observed that many *AtCLE* gene pairs, e.g., *AtCLE9*/*AtCLE10* and *AtCLE5*/*AtCLE6*, may have arisen through duplication. Additionally, many *AtCLE* genes were found in regions of the genome that were rich in repetitive sequences [48]. These results suggested that rearrangement and gene duplication were plausible mechanisms for the expansion of the *AtCLE* gene family [48]. Therefore, like *AtCLEs*, genome duplication and reshuffling contributed to the expansion of *PtCLE* gene family [48]. Moreover, unlike *Arabidopsis*, the subsequent diversity in the CLE motifs of PtCLEs also has driven the expansion of this family.

### Probing the roles of *PtCLE* genes by phylogenetic analyses and expression profiles between *Arabidopsis* and poplar

**Fig. 4:**
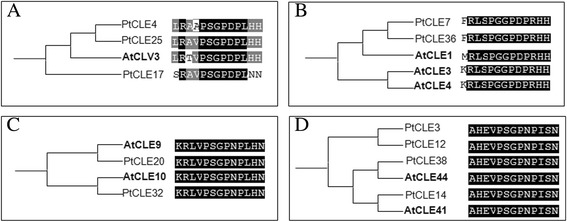
Comparative studies of representative AtCLEs and PtCLEs with identical or nearly identical CLE motifs. Each subclade which contains a well-known AtCLEs (bold) and their closest poplar counterparts derived from the phylogenetic analysis of all AtCLEs and PtCLEs as depicted in Additional files 10 and 11. The identical residues are shaded in black while similar residues are shaded in grey. **a** The AtCLV3 clade; **b** The AtCLE1 clade; **c** The AtCLE10 clade; **d** The AtCLE41/TDIF clade

As the first attempt to investigate potential role(s) of PtCLEs, the phylogenetic relationships between AtCLEs and PtCLEs were analyzed (Additional files 10 and 11). The phylogenetic analysis classified the PtCLEs and AtCLEs into several clades with varying degrees of phylogenetic distance based on the conserved CLE motifs that were used to construct the phylogenetic tree (Fig. 4; Additional file 10). Although the phylogenetic tree is based on the CLE motif alone, the clades defined by this tree correlated very well with phylogenetic relationship defined using full-length CLE proteins (Additional file 11).

Overall, our analysis indicated that the PtCLE proteins were quite closely related to their predicted *Arabidopsis* counterparts, which allowed interspecies identification of putative functional orthologs (Fig. 4; Additional files 10 and 11). Some clades segregated AtCLE and PtCLE proteins, whereas other clades contained CLE proteins of both species (Additional files 10 and 11). Each of these clades contained at least one functionally characterized member, allowing us to infer possible functions for the PtCLEs in the same clade (Additional files 10 and 11). Thus, the potential function of PtCLEs in each clade was predicted using functionally characterized AtCLEs [1–6, 49, 50]. As aforementioned, many PtCLE proteins contained perfectly matched CLE motifs (Additional file 9). Particularly, some PtCLE proteins comprised CLE motifs that matched completely with the CLE motifs of AtCLE proteins (Fig. 4; Additional file 9). It is presumed that PtCLEs with identical CLE motifs or PtCLEs carrying the same CLE motifs as that of AtCLEs might share similar protein functions [37, 40]. In addition to the CLE motif, the expression domain of *CLE* genes is also important for their functional specificities as have been shown that many AtCLE proteins acted interchangeably when ectopically expressed [37, 40, 45, 49, 51]. Therefore, we assessed potential roles of *PtCLE* genes using a combination of phylogenetic analyses and available transcriptomic data.

**Fig. 5:**
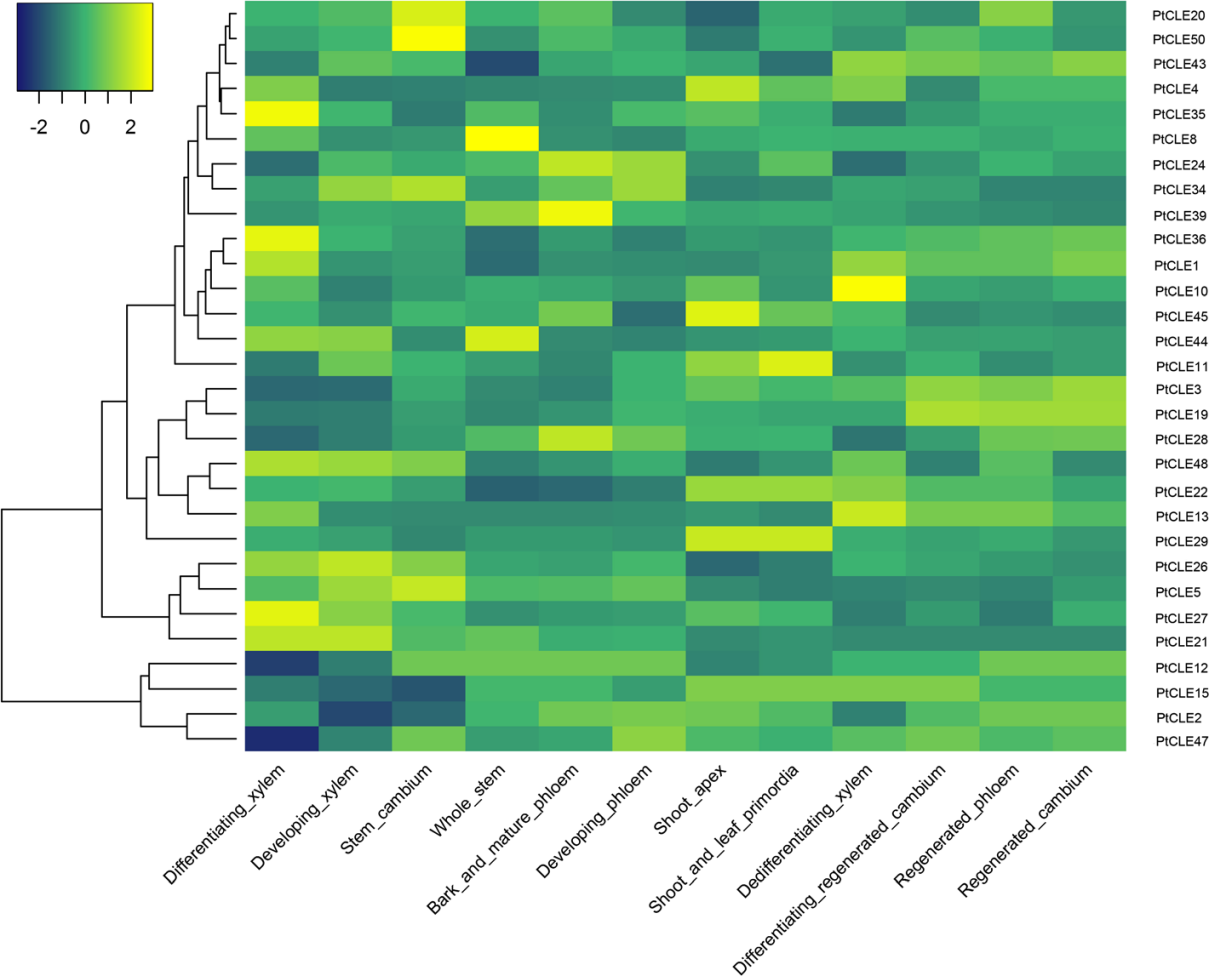
Transcriptional profiling of *PtCLE* genes in representative shoot and vascular tissues. The microarray data were downloaded from GEO and normalized for analysis. Color scale represents log2 expression values

The roles of AtCLV3, AtCLE1/AtCLE3/AtCLE4, AtCLE9/AtCLE10 and AtCLE41/TDIF have been functionally characterized previously [14, 15, 18, 19, 22, 23, 28, 33, 52]. We thus further identified PtCLEs sharing identical or nearly identical CLE motifs with those well-studied AtCLEs. Three PtCLEs (PtCLE4, PtCLE17 and PtCLE25) were grouped together with AtCLV3, of which PtCLE4 and PtCLE17 shared a nearly identical CLE motif with AtCLV3 (Fig. 4a; Additional files 10 and 11). AtCLV3, perceived by various parallel receptor complexes, restricted expression of the stem cell-promoting transcription factor WUS, which in turn activated *AtCLV3* expression, thus forming a negative feedback loop that maintained a balanced stem cell population [14, 15, 53–56]. Indeed, *PtCLE4* showed a highest expression level in shoot apex and a moderate expression level in shoot and leaf primordia of all materials tested, strongly supporting a possible role for *PtCLE4*, similar to AtCLV3, in regulating poplar shoot development (Fig. 5; Additional file 12). However, we cannot exclude the possibility that PtCLE17 and PtCLE25 also play roles in shoots.

*AtCLE1*, *AtCLE3* and *AtCLE4* repressed the lateral root development in a CLV1-dependent manner in *Arabidopsis* [28]. Two PtCLEs, PtCLE7 and PtCLE36, shared an identical CLE motif as that of AtCLE1/AtCLE3/AtCLE4 (Fig. 4b). *PtCLE36* was found to predominantly expressed in the xylem (Fig. 5; Additional file 12), unlike what has been observed for *AtCLE3* [28], suggesting a different role of PtCLE36. However, it still will be of great interest, as the first step, to investigate whether the expression of *PtCLE7* and *PtCLE36* are induced under nitrogen deficient conditions.

AtCLE9/AtCLE10 inhibited protoxylem vessel formation via CLV2 by repressing the expression of *ARR5* and *ARR6* in *Arabidopsis* roots [52]. A pair of PtCLEs (PtCLE20 and PtCLE32) comprised the same CLE motif as that of AtCLE9/AtCLE10 (Fig. 4c). Similar to *AtCLE10*, *PtCLE20* was highly expressed in vascular tissues (Fig. 5; Additional file 12). Numerous CLV2-like proteins have been mined from poplar [57], which favors the idea that a similar AtCLE9/AtCLE10-CLV2 signaling pathway regulates root vascular development in poplar as well.

A subclade of four PtCLEs (PtCLE3/PtCLE12/PtCLE14/PtCLE38) grouped together with AtCLE41/TDIF, sharing an identical CLE motif (Table 1; Fig. 4d; Additional file 3; Additional file 9), which strongly supported a conserved role of these peptides in the regulation of vascular cambium homeostasis in poplar and *Arabidopsis*. Intriguingly, in all materials tested, *PtCLE12* had the highest expression level in phloem, and was almost absent from xylem (Fig. 5; Additional file 12). This expression pattern was similar to that of its putative *Arabidopsis* counterpart *AtCLE41*/*TDIF* [19]. *PtCLE3*, another *PtCLE* gene encoding an identical CLE motif with that of AtCLE41/TDIF, was highly expressed in cambium and moderately expressed in phloem, which may suggest a broader role for PtCLE3 in poplar (Fig. 5; Additional file 12). In *Arabidopsis*, the plasma membrane-bound receptor PXY/TDR perceived the AtCLE41/TDIF to promote the (pro-)cambial proliferation by regulating *WOX4* expression, and to suppress (pro-)cambial cell differentiation into xylem cells [22, 23]. Recently, Etchells et al. [35] showed that tissue-specific expression of *PttPXY* and *PttCLE41* produced transgenic trees with increased wood production and a larger biomass. Notably, *PttCLE41* was the same CLE protein as PtCLE38 identified in this study (Table 1; Fig. 4f). Altogether, it seems possible that PtCLE12 also plays a similar role in the regulation of cambium development and wood formation. However, we cannot exclude the possibility that PtCLE14 carrying the same CLE motif as AtCLE41/TDIF, is also involved in (pro-)cambium stem cell homoeostasis (Fig. 5; Additional file 12). Taken all together, this pointed to the existence of a similar AtCLE41/TDIF-TDR/PXY module in regulating secondary growth in trees. However, whether the other three PtCLE proteins (PtCLE3/PtCLE12/PtCLE14), which contained an identical CLE motif as that of PttCLE41/PtCLE38, sharing a similar function remained unknown. Four hundred receptor-like kinases (RLKs) and eighteen WUS-related proteins have been identified in poplar, which supports the idea that the existence of multiple CLE-RLK-WOX signaling pathways [58, 59].

In summary, we grouped and compared the PtCLE proteins with their most closely-related AtCLE proteins to assess their potential roles based on functional studies [2–4, 6, 35, 49, 50]. The study indicated that PtCLE proteins are generally closely related to their predicted *Arabidopsis* counterparts. Intriguingly, many PtCLE proteins comprised exactly the same CLE motifs as that of their *Arabidopsis* counterparts, strongly suggesting functional conservation between specific AtCLEs and PtCLEs. However, It is also possible that those PtCLEs carrying identical CLE motifs play distinct roles in *planta* which could be achieved via tissue-specific expression pattern. Additionally, a few sets of PtCLEs shared an identical or nearly identical CLE motif, whereas no closely-related AtCLEs could be identified in the phylogenetic clades (Additional files 9, 10 and 11), raising the possibility that these PtCLEs may have unique functions in woody trees. It is of great interest to examine whether those PtCLEs possessing similar CLE motifs are functionally redundant as what has been observed in the *AtCLE* gene family [17, 26]. Nevertheless, it is important to assess to what extent these observations are supported by biological validation.

### Uncovering putative functions of *PtCLE* genes in shoot and vascular development

Previous studies have demonstrated that CLE peptides played various roles in plant growth and development [1–6]. To deepen our understanding of the potential functions of PtCLE proteins, *in silico* expression data for 30 out of 50 *PtCLE* genes were obtained from different *Populus* species other than *P. trichocarpa* for further analysis (Additional file 13). A total of six developmental microarray sets including samples derived from various organs and tissues were retrieved and normalized for further study with an emphasis on shoot organogenesis and vascular development (Additional file 13).

*PtCLE* genes generally exhibited differential expression patterns in the materials tested (Fig. 5; Additional files 12 and 13), similar to what was observed for the expression of *AtCLEs* [51]. Other than *PtCLE4*, *PtCLE11*, *PtCLE22*, *PtCLE29*, and *PtCLE45* are also highly expressed in shoot apex and/or shoot and leaf primordia (Fig. 5; Additional file 12). Among these, *PtCLE22* and *PtCLE29* showed consistent expression patterns in two tested samples. *PtCLE45* is limited to the shoot apex, whereas *PtCLE11* expression is relatively restricted to the shoot and leaf primordia, indicating a spatially and temporally expression fashion (Fig. 5; Additional file 12). However, whether any of these PtCLE proteins are involved in controlling the stem cell homoeostasis in shoot apex or in primordia remained to be proven.

The (pro-)cambium, a stem-cell tissue, gives rise to the phloem and xylem which perform essential roles in transportation of water, mineral nutrients and signaling molecules [60]. In *Arabidopsis*, the AtCLE41/TDIP-TDR/PXY-WOX4 signaling module plays an important role in (pro-)cambium proliferation and differentiation [19, 22, 23]. Additionally, a number of AtCLEs are shown to control vascular development, which assigned CLEs as central players mediating cell-cell communication in plant vascular development [5]. In our analysis, we found that a number of *PtCLE* genes are predominantly expressed in various vascular tissues, except the aforementioned *PtCLE3* and *PtCLE12* (Fig. 5; Additional file 12). *PtCLE5*, *PtCLE26* and *PtCLE34* are expressed at the highest level in cambium and xylem, while *PtCLE10/PtCLE13/PtCLE21/PtCLE27/PtCLE35/PtCLE36* exhibited a peak expression level in xylem. *PtCLE20* and *PtCLE50* are predominantly expressed in the cambium (Fig. 5; Additional file 12). The expression of *PtCLE24*, *PtCLE28* and *PtCLE39* is mainly detected in the phloem (Fig. 5; Additional file 12). The transcriptional activities of the remaining *PtCLE* genes are highly dynamic (Fig. 5; Additional file 12).

Interestingly, we found that *PtCLE* gene pairs encoding identical CLE motifs, including *PtCLE3*/*PtCLE12*, *PtCLE21*/*PtCLE39*, and *PtCLE28*/*PtCLE47*, exhibited both overlapping and distinct expression patterns with respect to different tissues (Fig. 5; Additional file 12). This points to functional divergence of these *PtCLE* genes in *planta* despite that they share the same CLE motif. We further investigated whether expression trends are similar between *AtCLE* genes and their putative poplar orthologues. In addition to previously high-resolution expression data for the entire *Arabidopsis* A-type *CLE* genes [51], we compiled and visualized the expression profile of *AtCLE* genes in selected tissues by e-Northerns browser of BAR (Additional file 14; [61]). *In silico* expression data for 14 out of 32 *AtCLE* genes were available. In the case of *AtCLE46*, it was highly expressed in meristematic tissues and xylem-rich samples (Additional file 14). A similar expression trend was observed for its putative poplar orthologues *PtCLE5* and *PtCLE26*, both of which exhibited significant expression levels in cambium and developing-/differentiating-xylem (Fig. 5; Additional file 12). However, only some *CLE* genes of *Arabidopsis* and poplar are presented in the microarrays, making it difficult for in-depth investigation. Nevertheless, it is also likely that other *PtCLE* genes which are not available on the microarrays show significant expression in some tissues. Thus we analyzed the available EST sequences and RNA-seq data to explore the expression of the *PtCLE* genes that are not presented in the microarray. The corresponding ESTs and RNA-seq reads were extracted from public databases, demonstrating that these *PtCLE* genes were transcribed based on the numbers of ESTs detected and the FPKM (the number of fragments per kilobase of exon per million fragments mapped) values for RNA-seq data (Additional file 15). In several cases, there are no matched EST(s) were identified in *P. trichocarpa*, but matched EST(s) from sibling species or high FPKM value could be detected (Additional file 15). The matched ESTs varied in numbers, suggesting that they are expressed differentially or the ones with few ESTs are probably expressed at low level or restrict to particular tissues or developmental stages. Altogether, our data indicated a complicated expression profile amongst the *PtCLE* genes, which is well correlated with their diverse roles in poplar growth and development.

## Conclusions

The *CLE* genes are well known for their roles in coordinating stem cell fate in different types of plant meristems including the vascular cambium, which is the most notable growth characteristic in tree species. In this study, the *CLE* gene family in *P. trichocarpa*, a tree species with extensive wood formation, was identified and classified into four major groups based on sequence similarity. The potential roles of *PtCLE* genes, with an emphasis on shoot organogenesis, secondary growth and wood formation, were analyzed by comparative studies and transcriptional profiling. A number of PtCLE proteins and their putative *Arabidopsis* orthologues were identified based on identical or nearly identical CLE motifs and comparable tissue expression expression patterns, pointing to possible functional conservation of these CLE proteins. Conversely, some *PtCLE* genes appeared to be regulated in completely different ways from their *Arabidopsis* counterparts, which may provide insights into the functional divergence of CLE signaling in tress species. The comparative studies further indicated close parallel regulation of AtCLEs and PtCLEs orthologues, which highlighted potential strategies such as manipulation of key plant peptide signaling molecules for higher yields and more sustainable wood sources.

## Methods

### Identification of PtCLE proteins and protein features analysis

All known CLE proteins were retrieved and used as queries to perform the BLASTP and TBLASTN programs searching against the *Populus trichocarpa* genome sequence (http://www.phytozome.net; [62]). Each identified hit subsequently was used as a new query to conduct a BLASTP search querying against the poplar assembly genomic sequence (Version 2.2) to avoid any missed PtCLE protein. The searches were run repeatedly until no new candidates were found.

SignalP (http://www.cbs.dtu.dk/services/SignalP), Multiple Expectation Maximization for Motif Elicitation (MEME) (http://meme.nbcr.net/meme/cgi-bin/meme.cgi) [63], and Weblogo (http://weblogo.berkeley.edu/logo.cgi) [64] were used for domain predictions and determination of domain features. SignalP was run for determining the signal peptides using both neural network (NN) and hidden Markov model (HMM) modes. In the cases that SignalP yielded low scores, the TargetP (http://www.cbs.dtu.dk/services/TargetP), iPSORT (http://ipsort.hgc.jp) and SecretomeP (http://www.cbs.dtu.dk/services/SecretomeP-2.0) were used to identify signal sequences.

### Genomic organization analysis

The exon/intron boundaries of each *PtCLE* genes were investigated using gene structure display server (http://gsds.cbi.pku.edu.cn) [65] and refined manually with expression data of EST sequences and cDNA sequences that were deposited in Phytozome (http://phytozome.jgi.doe.gov/pz/portal.html#!info?alias=Org_Ptrichocarpa). The chromosomal locations of *PtCLE* genes were determined using PopGenIE (http://popgenie.org/gp) [66].

### Alignment and phylogenetic analysis

Multiple alignments were performed using ClustalX [67], then refined and displayed using GeneDoc (http://www.psc.edu/biomed/genedoc). Phylogenetic trees were constructed by MEGA5 using either the conserved CLE motifs or full-length CLE proteins [68]. Bootstrap analysis was conducted with 1000 replicates to verify the significance of nodes.

### Gene expression analysis

Microarray data were obtained from the Gene Expression Omnibus database (GEO) at NCBI website. As a result, six developmental microarray datasets were collected as shown in Additional file 13. The downloaded raw CEL files were analyzed using the Affy package in R language [69], followed by the background correction and microarray expression normalization using the RMA method [70]. Differential gene expression was determined according to [71], which was followed by a multiple testing correction [72]. Heatmaps were generated based on the expression profiles, in which cluster of PtCLE proteins were determined as well. The EST (Expressed Sequence Tags) sequences and RNA-seq data were obtained from Phytozome. Transcript abundances based on RNA-Seq data in mixed tissues were calculated as numbers of fragments per kilobase of exon in a gene per million fragments mapped (FPKM).

### Availability of supporting data

Phylogenetic data have been deposited to TreeBase and are accessible via the URL: http://purl.org/phylo/treebase/phylows/study/TB2:S18866. Additional supporting data are included as additional files.

## Additional files

Additional file 1: A list of full-length sequences of all PtCLE proteins. The signal peptide cleavage sites of every PtCLEs are indicated. (PDF 27 kb) Additional file 2: The multiple alignment of all full-length PtCLE proteins. The C-terminal CLE motifs of each PtCLE were boxed. (PDF 38 kb) Additional file 3: The multiple sequence alignment of the CLE motifs derived from PtCLE proteins. The conserved residues are shaded in grey. (PDF 28 kb) Additional file 4: The multiple sequence alignment of all AtCLE and PtCLE proteins using their full-length proteins. The CLE motifs were boxed in red. The conserved residues are shaded in grey. (PDF 46 kb) Additional file 5: The multiple sequence alignment of all AtCLE and PtCLE proteins using their CLE motifs. The conserved residues are shaded in grey. (PDF 14 kb) Additional file 6: The multiple sequence alignment of all PtCLE proteins using their CLE motifs and five N-terminal residues flanking the CLE motifs (18-AA in length). The conserved residues are shaded in grey. Weblogo plot was used for graphical representation of the multiple sequence alignment of the 18-AA fragments. (PDF 72 kb) Additional file 7: The multiple sequence alignment of all AtCLE and PtCLE proteins using their CLE motifs and five N-terminal residues flanking the CLE motifs (18-AA in length). The conserved residues are shaded in grey. Weblogo plot was used for graphical representation of the multiple sequence alignment of the 18-AA fragments. (PDF 58 kb) Additional file 8: Phylogenetic analysis of PtCLE proteins by the Neighbor-joining method with 1000 bootstrap iterations. The tree was constructed using full-length PtCLE proteins. The percentage of trees in which the associated clades clustered together is shown (>40 %). (PDF 107 kb) Additional file 9: A list of AtCLE and PtCLE proteins with identical CLE motifs. (PDF 12 kb) Additional file 10: Phylogenetic analysis of AtCLE and PtCLE proteins by the Neighbor-joining method with 1000 bootstrap iterations. The tree was constructed using the conserved CLE motifs. The percentage of trees in which the associated clades clustered together is shown (>40 %). (PDF 70 kb) Additional file 11: Phylogenetic analysis of AtCLE and PtCLE proteins by the Neighbor-joining method with 1000 bootstrap iterations. The tree was constructed using full-length proteins. The percentage of trees in which the associated clades clustered together is shown (>40 %). (PDF 39 kb) Additional file 12: Transctiptional profiling of *PtCLE* genes in various organs and tissues using the available microarray data. The microarray data were downloaded from GEO and normalized for analysis. Color scale represents log2 expression values. (PDF 2632 kb) Additional file 13: A list of microarray datasets used in this study. Note that available microarray data were derived from different *Populus* species other than *P. trichocarpa*. (PDF 31 kb) Additional file 14: The expression pattern of *AtCLE* genes in shoot- and vascular-related tissues. Gene expression is displayed as normalized log2-transformed values. (PDF 148 kb) Additional file 15: The EST sequences and RNA-seq data for *PtCLE* genes which are not presented in the microarray. The expression level for RNA-seq data was presented as numbers of fragments per kilobase of exon in a gene per million fragments mapped (FPKM). (PDF 237 kb)
