# Supplementary material for: Identification and characterization of the Populus trichocarpa CLE family
Source: BMC Genomics. 2016 Mar 2;17:174. doi: 10.1186/s12864-016-2504-x (PMC4776436; doi:10.1186/s12864-016-2504-x)
Supplement: Additional file 1: — A list of full-length sequences of all PtCLE proteins. The signal peptide cleavage sites of every PtCLEs are indicated. (PDF 27 kb) [file 12864_2016_2504_MOESM1_ESM.pdf]

>PtCLE1
MAFGDGLSRFSSISIALIILTFVLM**PL**VHSSRSSSLNIRFTNMACNLIMFAMHET
TVAAGNRARASAQDIHQSYKITRMGKGASSLVTDEESEREVPTGPDPLHHNN
NPTTRP

>PtCLE2
MDIDPFWITGGWLIITDFNFMAPTRKS**PL**CRTTTKLQAMVLFLDLLLIVPLLAR
PIDLSKKLTAASSPTGTKHSTTEMHPHESKYAPPSSTTAAAAAADMTSSTTTTV
PTTPAASASNQQFKAAAHEVPSGPNPESN

>PtCLE3
MAIKEHRFLLGTSRDGEYIKKNDMEYF**AN**RRHDMGNAKTVSKANIIHIPPPSS
RRRGRFRAHRSPLPWQEGVFNDSAHEVPSGPNPISNRKNQKQLEKERCAKYI
KREMSFHRQGKGITISTFGVRRPFVKDETKYNYERETKGPKYQSFACKVTRT
WHPKL

>PtCLE4
MACPSKFYSLMLVLVLFYMVMEESY**GL**NLSQSLSLHGCSTGQRCFYAEAVSP
VDVKSRKVLVVLTGGLRGPTGSTGNGEKLEIRELRAAPSGPDPLHHNGGSPEK
PRTP

>PtCLE5
MKWIKIASCLSRTLSSFSRSMQHFKPRMSRLTFVHLLLAWLLLVA**SQ**QRFSSNI
KVQAVEAVHFKPNPAQLTSKSHKGNVLPVWVAEKRIHKSPSGPNPVGNHNPPS
KQ

>PtCLE6
MRQLMGLRKELACLAIIFFILLLLETS**SL**PDRSARYGSSKNTGSTSQLMGPVKS
HGGGLRGDKDEGGDATLGDEKRKIFTGPNPLHNR

>PtCLE7
MANMRVFLFFILSLLFFSTFE**TR**SIDRISHRGDRSLIETAQEMLKESIARHELIEG
FNESFRLSPGGPDPRHH

>PtCLE8
MATLIKKKILLLSMIIIVMLVGSSD**AR**FSRKFSTMPKKFESSHILRELGYDMPKI
EYYRRRWMLDTDRLSPGGPDPQHH

>PtCLE9
MHYQQSPRVFVLQKPMVILLLVLFVS**GD**RKEETTVNVHGLQNQQPPSSTEKQ
QRLRHSFDTFFSSKRKVPNASDPLHNR

>PtCLE10
MKLFQLCLIVALLIVFGSSPRRSH**AI**RGSSSAPSTSQQVFRSPFSPSPFAQRAEEF

ASQKRRVPAGPNPLHNKR

>PtCLE11
MGTTTLYPRLSFFVLMIMLVVNQLSSCHFIHSRTSEEPGKTVETDLSSHFSWKF
KEMVRERSSKDESDTIYRVSRRKIPAGPNPLHN

>PtCLE12
MDIEPLWALGGWFLFSITCMATPKSQSTISETFKRSHHFFLFLALLFVFILLTSPS
KPINPTNTVASISIKRLLLESSEPASTTMNLHPKHTQGTRTSSSSSSPPSSKSTRK
KFGAQAHEVPSGPNPISNR

>PtCLE13
MRINPNPRLSFIFLIIIILAISQLSSCRHLHIKIGDQNKQRAEADVFTQLSWHFPA
KASEGSSKDEIDDPVYGVSYRAVPGGPNPLHN

>PtCLE14
MASDVGSPNLTSLTILFFLLIMFHTTMANKDHRFLLSTTRDGGYFKKSLMEFS
TTRPDMGNAKTVSKANVIHIPPQSSRRRGRFRAHRSPLPWQEGIFSASAHEVP
SGPNPISNR

>PtCLE15
MDIDPFWITGGWFIITDFNFMAPARQLSVCENTTKLQAMVLFLGLLLIVPLLA
RPIDLSKKFTASSPSTRAKHFTTEIHPQESKYTPPSRTTDDAAAVTSSTTTTVSA
TPVSASRQQFKAAFHEVPSGPNPESN

>PtCLE16
MASLRCYLCVLLILLSFAQSEARPLDPSVVRRNLIRTIRALGESEAVGDQCASK
RVSPGGPDAKHH

>PtCLE17
MFTSKNKVGYLLLILLSISAFDHGVLGARNLKERVEYTKKTEGSVNIQDATVS
RAVPSGPDPLNNR

>PtCLE18
MINRVTRKKEMGVLRRELACLSLLLLLLLIMSLLETPCYAVGYGKFSSVKGGS
SSELRNNPAMSNSVGGLKRNANKDGNEIFGADKRKVYTGPNPLHNR

>PtCLE19
MVFCAHRVLILLICIGFIAVQPDEVYGLTSVELVLRHNQKAQGTAPQSQRVLK
DVDMQGMDTKKSAHASKTFDRSQSNKRRAPRGSDPIHNRERDVYRKAIREN
VHRASIISVPQVEIIINRQ

>PtCLE20
MKNPFSSTTTLSSHGLLILALLLLFFVISSTATGVPTSASLETSSRNQHHRFKSQ

HHSCGSFPHKSSSRSWCIRFQRMNGRRHLGSPLPPPLPPPIEIDPRYGVEKRLVP
SGPNPLHN

>PtCLE21
MALKISHIPCALLCLYLLLL**AF**HELRNFKSKINNNKDQINNISSSSISHHPFHNR
KVLVSKFDFTPFQKHRQQQHENPLPDEEVHKKAARSEIDPRYGVEKRLVPTGP
NPLHH

>PtCLE22
MSFGSSRRIMYSTSLVVVLVMFVLQIWVC**GV**SNCKAGAIRLLQENDMAKFKE
SGNNIPANNYKSKEEYFRKYFNERGNTSHGFNKTEKGFEESKRRVPSCPDPLH
N

>PtCLE23
MIFHRKVGVAAARGRRYSGAKAAIIFLFWILLILAQLGLLIAFGHEETGKLVKS
LPRKARFFETRFHAPPSQDQPLDIDKGDPDTVYEDDKRIIHTGPNPLHN

>PtCLE24
MGGGGSCSLSFKVLLAAVATVMLVLLLLVGALESGATKMTERTQTVLDSIAQ
DDLRRRHEELIGREKLVYNPELDLNFVMNKRKVPNGPDPIHNRRAGNSRRPP
GRA

>PtCLE25
MALSFKFYSLIFLLGLLFMVLEESS**GC**KTGEKCFYGDAASLVDFKSRKVLVVS
RGDVRGEPTSNSTGNGEELEIRELRAVPSGPDPLHHNGGSPKKPRTP

>PtCLE26
MITDNSVDKNTPYSSKPLSHKASPFICNTHPFKPKMSRLTFTIHLLLALLLLVAS
QQHFPSIIKVQAIEAVHFKLKPRQLTSKLHVGDDLPNWVAEKRIHKSSSGPNPV
GNRNPPSKQ

>PtCLE27
MGLRKELACLALLFLILLLLETS**SV**PDRSARHGSFKNTGSTAQLTGPVKSHGG
GLRGDRDEEGDATLGDEKRKVYTGPNPLHNR

>PtCLE28
MGCSGSSCSLSFKVLLGGIATVVFVMLLLVGALESGATSKMTTSRLNSVQATQ
NDLKDDHEKDVIGREKLVYNSELDLNYMMSKRRVPNGPDPIHNRRAGNSKR
PPGRA

>PtCLE29
MVSHHKVGVEAARARRYAGAARAAII**FL**FWILLILAQLGV**FL**VVHEETDKPV
KSLPRKARVFETGSVHASPNQDQPVNIDGGDPDAVYEDDKRTIHTGPNPLHN

>PtCLE30
MSFGSSRRLMYSSSLVVVLVVFVLQIWVC**SD**CNCKAGAIRLLQENGMEKFKE
SSDITKDNYKSKEKHFRKYFNERANTSYGFNKTEKGFEENKRRVPSCPDPLHN

>PtCLE31
MALRIFHTLCVVFWLSLLLLVF**HE**LCNFKSKINSTKDQINNISKSSTFSYRPFLT
RKVVARKFDFTPFQKHHQQPMPEEEGHKKRARSEIDPRYGVEKRLVPTGPNP
LHH

>PtCLE32
MLGIKTPSLLSPPSFPFSFLSKPLSLQHSPS**PF**DLSISLPTMKNPLSSTISFSSQYR
LLILTLLLFFVISTATRIPNYASLDTSSRNHRDSFKIQRYSPSSFPRKSTSSYWCN
QFQRMKGGLHLGPPPPPPPPSEIDPIYGVEKRLVPSGPNPLHN

>PtCLE33
MVFCSRRVLILLICIGFIAVQ**PR**KLYGLTSVEVILRHDRKAHGTLPHSQRSLKD
VDMQGMDTKKSAQANKTFDPSQTSKRRVRRGSDPIHNRS

>PtCLE34
MKRKQILAYALLAFLIASDQCHYSA**GI**VVQAAKSVDTRLKNAQPILRSTRYKL
ASWKSGTKFKDTIHKAPSGPSPIGNRHRSSIHV

>PtCLE35
MACVRFYLCVMLILLSFAQ**SE**TRPLDPSAVRRNLIRTIRALGETETYNVKQGN
EGMIGGRFSSKRVSPGGPDAQHH

>PtCLE36
MANMRLLLFFVLIMLLFSMFE**TR**SIDHVAHRRDRSLIESSKEMVKESIVRHEM
TGGFNECFRLSPGGPDPRHH

>PtCLE37
MATSMKMRILLLSILLLLMLVGSSD**AR**FSRKFSIMPEKLVSRHILRDLGYEMSK
VEHYRRWMQDTDRVSPGGPDPHHH

>PtCLE38
MATPKTQSTTISDHQTCTKAHH**FL**SLL**AL**LFIFILLTTSTKPINPTNMAASISIKR
LLLESSEPASTTMNLHPKQTQDARTSSSSSSSTSSSKSTRTKFGAAAHEVPSGP
NPISNR

>PtCLE39
MNIAVKIPQYSFSIIAWLFLF**LF**FFHGWCYFFLNSNNIIHNNNNVHVSPRTLSNR
KMLVSNVDFTAIMSRHHQRHMPMHSDPTRGEIDPLYGVEKRLVPTGPNPLHH

>PtCLE40
MRTYSPLLPLLVFSTILLASLHSST**CR**HVSWTTYEEKQQINTKYPLPFPQYDLP
GISHTVKSKDDKVNKLFGGSHKAVPGGPNPLHN

>PtCLE41
MKNKNSQLFLIFLMVFLVLVH**GT**TCRDIKRSIGNGEIEQGSETKHSSTFLQARS
AIFKASESSNNKIKEVHSVSRRLVPSGPNPLHN

>PtCLE42
MFMSRSQVGCLLLVLLSISAFHHGVV**GA**RNLKENVEAKEKTQNEKEGADSE
DINGYVANVNRVVPSCPDPIHNR

>PtCLE43
MANLKFWVCLVLLFLTLAM**SE**TRHLDQPYLGRKNLARILQELQEKSKQVDV
RFIDDGDVARSPYESKRLSPGGPDPKHH

>PtCLE44
MKLFHLFLLLALLIFFSSTPRSSH**AA**RRSFSAPSTSQQVFRSPFRASPFAERAKE
FESQKRKVPTGSNPLHNKR

>PtCLE45
MRIAYSPLLPLLVFSTIMLSLLHPST**CR**HISRATYEEEQQLNTEFSLPLPQHLPAI
AHTVKFNKDDKVQKLYAASHKLVPGGPNPLHN

>PtCLE46
MGIGQCNLAALHIHCLFERQL**QN**AFLNKNILLQSTNNKPTIFPSALKSIFLSINL
SGLYIPTVFIMRNKNPQLFLIFLIVFLVLVHGTTCRDTKRSTSNGETEQGSKTKH
SSMFLQALSSIFKASESSTNNIKALHTVSRRLVPCGPNPLHN

>PtCLE47
MGRGGRILRALLGAVIFWGVIWFLYV**GI**LPNHATTLMARIRVPAAGTFQHLKL
SGRESHLIRHDMDLNYVSKRRVPNGPDPIHNRKTVQSRQPPGQS

>PtCLE48
MASRVASTSRAMILLLMVLFSAIFLTSE**AR**ILKGGQALQGNANNSRHLLLELG
FDLSKLEHYRRLSTLSVASDRLSPGGPDPHHH

>PtCLE49
MANIPTATRVLVVLLVVFMMLMRFE**AA**PIHTLKELDKRLLLSKVLNAKSRME
FHGRRMSISESATDRLSPEGPNHEHHSHPPGNP

>PtCLE50
MANLKLWVCLLLLFLTFSKSE**TR**LLDQPYLGRKNPARMLQELNEKSKQLFED
DSDVTGSPYEPKRISPGGPDPKHH
