# Supplementary material for: Identification and characterization of the Populus trichocarpa CLE family
Source: BMC Genomics. 2016 Mar 2;17:174. doi: 10.1186/s12864-016-2504-x (PMC4776436; doi:10.1186/s12864-016-2504-x)
Supplement: Additional file 2: — The multiple alignment of all full-length PtCLE proteins. The C-terminal CLE motifs of each PtCLE were boxed. (PDF 38 kb) [file 12864_2016_2504_MOESM2_ESM.pdf]

```
PtCLE40: -------------------------------------------------------------------MR-TYSPLLPLLVFSTILLASLHSSTCRHV-------SWTTY--EEKQQINTKYPLPFPQYDLPG
PtCLE45: -------------------------------------------------------------------MRIAYSPLLPLLVFSTIMLSLLHPSTCRHI-------SRATY--EEEQQLNTEFSLPLPQH-LPA
PtCLE41: -------------------------------------------------------------------MK-NKNSQL-FLIFLMVFLVLVHGTTCRDI-------KRSIGNGEIEQGSETKHSSTFLQA-RSA
PtCLE46: MGIGQCNLAALHIHCLFERQLQNAFLNKNILLQSTNNKPTIFPSALKSIFLSINLSGLYIPTVFIMR-NKNPQL-FLIFLIVFLVLVHGTTCRDT-------KRSTSNGETEQGSKTKHSSMFLQA-LSS
PtCLE2 : -----------------------------------MDIDPFWITGGWLIITDFNFMAPTRKS---PLCRTTTKLQAMVLFLDLLLIVPLLARPIDL-------SKKLTAASSPTGTKHSTTEMHPHESKYA
PtCLE15: -----------------------------------MDIDPFWITGGWFIITDFNFMAPARQL---SVCENTTKLQAMVLFLGLLLIVPLLARPIDL-------SKKFTASSPSTRAKHFTTEIHPQESKYT
PtCLE12: -----------------------------------MDIEPLWALGGWFLFSITCMATPKSQS-TIS--ETFKRSHHFFLFLALLFVFILLTSPSKP-------INPTNTVASISIKRLLLESSEPASTTMN
PtCLE38: -------------------------------------------------------MATPKTQSTTISDHQTCTKAHHFLSLLALLFIFILLTTSTKP-------INPTNMAASISIKRLLLESSEPASTTMN
PtCLE3 : --------------------------------------------------------------------------------MAIKEHRFLLGTSRDGE-------YIKKNDMEYFANRRHDMGNAKTVSKANI
PtCLE14: -----------------------------------------------------MASDVGSPNLTSLTILFFLLIMFHTTMANKDHRFLLSTTRDGG--------YFKKSLME-FSTTRPDMGNAKTVSKANV
PtCLE5 : -----------------------------------------------------------------MKWIKIAS-CLSRTLSSFSRSMQHFKPRMSRLTF--------VHLLLAWLLLVASQQRFSSNIKVQAVEA
PtCLE26: -------------------------------------------------------MITDNSVDKNTPYSSKPLSHKASPFICNTHPFKPKMSRLTFT-------IHLLLALLLLVASQQHFPSIIKVQAIEA
PtCLE10: -------------------------------------------------------------------------MKLFQLCLIVALLIVFGSSPRRSH------------------AIRGSSSAPSTSQQVFR
PtCLE44: -------------------------------------------------------------------------MKLFHLFLLLALLIFFSSTPRSSH------------------AARRSFSAPSTSQQVFR
PtCLE6 : -------------------------------------------------------------------MRQLMG-LRKELACLAIIFFILLLLETSS-----------------LPDRSARYGSSKNT--GST
PtCLE27: -------------------------------------------------------------------------MG-LRKELACLALLFLILLLLETSS-----------------VPDRSARHGSFKNT--GST
PtCLE18: -------------------------------------------------------------MINRVTRKKEMGVLRRELACLSLLLLLLIMSLLE-----------------TPCYAVGYGKFSSVKGGSS
PtCLE20: -------------------------------------------------------MKNPFSSTTTLSSHGLLILALLLLFFVISSTATGVPTSASLE-------TSSRNQHHRFKSQHHSCGSFPHKSSSRS
PtCLE32: ------------MLGIKTPSLLSPPSFPFSFLSKPLSLQHSPSPFDLSISLPTMKNPLSSTISFSSQYRLLILTLLLFFVIS-TATRIPNYASLD-------TSSRNHRDSFKIQRYSPSSFPRKSTSSY
PtCLE21: -------------------------------------------------------------MALKI-SHIPCALLCLYLLLLAFHELRNFKSKINNN-------KDQINNIS-SSISHHPFHNRKVLVSKF
PtCLE31: -------------------------------------------------------------MALRI-FHTLCVVFWLSLLLLVFHELCNFKSKINST-------KDQINNISKSSTFSYRPFLTRKVVARKF
PtCLE39: -----------------------------------------------------------MNIAVKIPQYSFSIIAWLFLFLFFFHGWCYFFLNSNNI-------IHNNNNVH----VSPRTLSNRKMLVSNV
PtCLE11: -----------------------------------------------------------MGTTTLYPR-------LSF-FVLMIMLVVNQLSSCHFI-------HSRTSE-----EPGK--TVETDLSSHFS
PtCLE13: -----------------------------------------------------------MRIN-PNPR-------LSFIFLIIIILAISQLSSCRHL-------HIKIGD-----QNKQ--RAEADVFTQLS
PtCLE23: -----------------------------------------------------MIFHRKVGVAAARGRRYSG-AKAAIIFLFWILLILAQLGLLIAF-------GHEETG-----KLVKSLPRKARFFET-R
PtCLE29: -----------------------------------------------------MVSHHKVGVEAARARRYAGAARAAIIFLFWILLILAQLGVFLVV--------HEETD-----KPVKSLPRKARVFETGS
PtCLE22: -------------------------------------------------------------MSFGSSRR----IMYSTSLVVVLVMFVLQIWVCGVSNCKAGAIRLLQENDMAKFKESGNNIPANNYKSKEE
PtCLE30: -------------------------------------------------------------MSFGSSRR----LMYSSSLVVVLVVFVLQIWVCSDCNCKAGAIRLLQENGMEKFKES-SDITKDNYKSKEK
PtCLE4 : ----------------------------------------------------------------MACPS----KFYSLMLVLVLFYMVMEESYG--LNLSQSLSLHGCSTGQRCFYAE--AVSPVDVKSRKV
PtCLE25: ----------------------------------------------------------------MALSF----KFYSLIFLLGLLFMVLEESS------------GCKTGEKCFYGD--AASLVDFKSRKV
PtCLE17: ---------------------------------------------------------------------------MFTSKNKVGYLLLIL-------------------LSISAFDHG--VLGARNLKER--
PtCLE42: ---------------------------------------------------------------------------MFMSRSQVGCLLLVL-------------------LSISAFHHG--VVGARNLKEN--
PtCLE19: ----------------------------------------------------------------MVFCAH----RVLILLICIGFIAVQPDEVYG-----------LTSVELVLRHNQKAQGTAPQS-QRVL
PtCLE33: ----------------------------------------------------------------MVFCSR----RVLILLICIGFIAVQPRKLYG-----------LTSVEVILRHDRKAHGTLPHS-QRSL
PtCLE24: ----------------------------------------------------------------MGGGGS-CSLSFKVLLAAVATVMLVLLLVG-----------ALESGAT-KMTERTQTVLDSIAQDDL
PtCLE28: ----------------------------------------------------------------MGCSGSSCSLSFKVLLGGIATVVFVMLLLVG-----------ALESGATSKMTTSRLNSVQAT-QNDL
PtCLE47: ----------------------------------------------------------------MGRGGR----ILRALLGAVIFWGVIWFLYVG-----------ILPNHATTLMARIRVPAAGTF--QHL
PtCLE7 : -----------------------------------------------------------------MAN-------MRVFLFFILSLLFFS------------------TFETRSIDRISHRGDRSLIE--T
PtCLE36: -----------------------------------------------------------------MAN-------MRLLLFFVLIMLLFS------------------MFETRSIDHVAHRRDRSLIE--S
PtCLE16: -----------------------------------------------------------------MAS-------LRCYLCVLLILLSFA------------------QSEARPLD-PSVVR-RNLIR--T
PtCLE35: -----------------------------------------------------------------MAC-------VRFYLCVMLILLSFA------------------QSETRPLD-PSAVR-RNLIR--T
PtCLE43: -----------------------------------------------------------------MAN-------LKFWVCLVLLFLTLA------------------MSETRHLDQPYLGR-KNLAR--I
PtCLE50: -----------------------------------------------------------------MAN-------LKLWVCLLLLFLTFS------------------KSETRLLDQPYLGR-KNPAR--M
PtCLE8 : -----------------------------------------------------------------MAT---LIKKKILLLSMIIIVMLVG------------------SSDARFSRKFSTMP-KKFESSHI
PtCLE37: -----------------------------------------------------------------MAT---SMKMRILLLSILLLLMLVG------------------SSDARFSRKFSIMP-EKLVSRHI
PtCLE48: -----------------------------------------------------------------MASRVASTSRAMILLLMVLFSAIFL------------------TSEARILKGGQALQGNANNSRHL
PtCLE34: --------------------------------------------------------------------------MKRKQILAYALLAFLIASDQCHYS---------AGIVVQAAKSVDTRLKNAQPILRSTR
PtCLE9 : ---------------------------------------------------------------MHYQQS---PRVFVLQKPMVILLLVLFVSGD---------------------RKEETTVNVHGLQNQQP
PtCLE49: -----------------------------------------------------------------MAN---IPTATRVLVVLLVVFMMLMR------------------FEAAPIHTLKELDKRLLLSKVL
PtCLE1 : ----------------------------------------------------------------MAFGDGLSRFSSISIALIILTFVLMPLVHSSRSS-------SLNIRFTNMACNLIMFAMHETTVAAGNR
```

```
PtCLE40: ISHTVK-SKDDK------------------VNKLFGGSHKAVPGGPNPLHN-----------------------------------------------------------------------
PtCLE45: IAHTVKFNKDDK------------------VQKLYAASHKLVPGGPNPLHN-----------------------------------------------------------------------
PtCLE41: IFKASE-SSNNK------------------IKEVHSVSRRLVPSGPNPLHN-----------------------------------------------------------------------
PtCLE46: IFKASE-SSTNN------------------IKALHTVSRRLVPCGPNPLHN-----------------------------------------------------------------------
PtCLE2 : PPSSTTAAAAAADMTSSTTTTVPTTPAASASNQQFKAAAHEVPSGPNPESN-----------------------------------------------------------------------
PtCLE15: PPSRTTDDAAA--VTSSTTTTVSATP-VSASRQQFKAAFHEVPSGPNPESN-----------------------------------------------------------------------
PtCLE12: LHPKHTQGTRT----SSSSSSPPSS---KSTRKKFGAQAHEVPSGPNPISNR----------------------------------------------------------------------
PtCLE38: LHPKQTQDART----SSSSSSSTSSS--KSTRTKFGAAAHEVPSGPNPISNR----------------------------------------------------------------------
PtCLE3 : IHIPPPSSRRR----GRFRAHRSPLP---WQEGVFNDSAHEVPSGPNPISNRKNQKQLEKERCAKYIKREMSFHRQGKGITISTFGVRRPFVKDETKYNYERETKGPKYQSFACKVTRTWHPKL
PtCLE14: IHIPPQSSRRR----GRFRAHRSPLP---WQEGIFSASAHEVPSGPNPISNR----------------------------------------------------------------------
PtCLE5 : VHFKPNPAQLT----SKSHKGNVLPV---WVA---EKRIHKSPSGPNPVGNHNPPSKQ------------------------------------------------------------------
PtCLE26: VHFKLKPRQLT----SKLHVGDDLPN---WVA---EKRIHKSSSGPNPVGNRNPPSKQ------------------------------------------------------------------
PtCLE10: SPFSPSPFAQR--------------------AEEFASQKRRVPAGPNPLHNKR---------------------------------------------------------------------
PtCLE44: SPFRASPFAER--------------------AKEFESQKRKVPTGSNPLHNKR---------------------------------------------------------------------
PtCLE6 : SQLMG-PVKSHG---GGLRGDKDEGG-----DATLGDEKRKIFTGPNPLHNR----------------------------------------------------------------------
PtCLE27: AQLTG-PVKSHG---GGLRGDRDEEG-----DATLGDEKRKVYTGPNPLHNR----------------------------------------------------------------------
PtCLE18: SELRNNPAMSNSV--GGLKRNANKDG-----NEIFGADKRKVYTGPNPLHNR----------------------------------------------------------------------
PtCLE20: WCIRFQRMNGRR---HLGSPLPPPLPPPIEIDPRYGVEKRLVPSGPNPLHN-----------------------------------------------------------------------
PtCLE32: WCNQFQRMKGGL---HLGP--PPPPPPPSEIDPIYGVEKRLVPSGPNPLHN-----------------------------------------------------------------------
PtCLE21: DFTPFQKHRQQHENPLPDEEVHKKAARSEIDPRYGVEKRLVPTGPNPLHH-----------------------------------------------------------------------
PtCLE31: DFTPFQKHHQQ----PMPEEEGHKKRARSEIDPRYGVEKRLVPTGPNPLHH-----------------------------------------------------------------------
PtCLE39: DFTAIMSRHHQR---HMP---MHSDPTRGEIDPLYGVEKRLVPTGPNPLHH-----------------------------------------------------------------------
PtCLE11: WKFKEMVRERS---------SKDE------SDTIYRVSRRKIPAGPNPLHN-----------------------------------------------------------------------
PtCLE13: WHFPAKASEGS---------SKDEI-----DDPVYGVSYRAVPGGPNPLHN-----------------------------------------------------------------------
PtCLE23: FHAPPSQDQPL---------DIDKGD----PDTVYEDDKRIIHTGPNPLHN-----------------------------------------------------------------------
PtCLE29: VHASPNQDQPV---------NIDGGD----PDAVYEDDKRTIHTGPNPLHN-----------------------------------------------------------------------
PtCLE22: YFRKYFNERGN------------TSHGFNKTEKGFEESK-RRVPSCPDPLHN-----------------------------------------------------------------------
PtCLE30: HFRKYFNERAN------------TSYGFNKTEKGFEENK-RRVPSCPDPLHN-----------------------------------------------------------------------
PtCLE4 : LVVLTGGLRG------------PTG-STGNGEKLEIRELRAAPSGPDPLHHNGGSPEKPRTP-------------------------------------------------------------
PtCLE25: LVVSRGDVRGE-----------PTSNSTGNGEELEIRELRAVPSGPDPLHHNGGSPKKPRTP-------------------------------------------------------------
PtCLE17: -VEYTKKT----------------EGS----VNIQDATVSRAVPSGPDPLNNR----------------------------------------------------------------------
PtCLE42: -VEAKEKTQNE-----------KEGADSEDINGYVANVNRVVPSCPDPIHNR----------------------------------------------------------------------
PtCLE19: KDVDMQGMDTK-----------KSAHASKTFDRSQSN-KRRAPRPSDPIHNRERDVYRKAIRENVHRASIISVPQVEIIINRQ------------------------------------------
PtCLE33: KDVDMQGMDTK-----------KSAQANKTFDPSQTS-KRRVRRPSDPIHNRS---------------------------------------------------------------------
PtCLE24: RRRHE-ELIGR-----------EKLVYNPELDLNFVMNKRKVPNGPDPIHNRRAGNSRRPPGRA-------------------------------------------------------------
PtCLE28: KDDHEKDVIGR-----------EKLVYNSELDLNYMMSKRKVPNGPDPIHNRRAGNSKRPPGRA-------------------------------------------------------------
PtCLE47: K------LSGR-----------ESHLIRHDMDLNYVS-KRRVPNGPDPIHNRKTVQSRQPPGQS-------------------------------------------------------------
PtCLE7 : AQEMLKESIAR-----------------HELIEGFNESFRLSPGGPDPRHH-----------------------------------------------------------------------
PtCLE36: SKEMVKESIVR-----------------HEMTGGFNECFRLSPGGPDPRHH-----------------------------------------------------------------------
PtCLE16: IRALGESE----------------------AVGDQCASKRVSPGGPDAKHH-----------------------------------------------------------------------
PtCLE35: IRALGETETYN-----------VKQGNEGMIGGRFSSKRVSPGGPDAQHH-----------------------------------------------------------------------
PtCLE43: LQELQEKSKQVD----------VRFIDDGDVARSPYESKRLSPGGPDPKHH-----------------------------------------------------------------------
PtCLE50: LQELNEKSKQL-------------FEDDSDVTGSPYEPKRISPGGPDPKHH-----------------------------------------------------------------------
PtCLE8 : LRELGYDMPKI--------------EYYRRR--WMLDTDRLSPGGPDPQHH-----------------------------------------------------------------------
PtCLE37: LRDLGYEMSKV--------------EHYRR---WMQDTDRVSPGGPDPHHH-----------------------------------------------------------------------
PtCLE48: LLELGFDLSKL--------------EHYRRLSTLSVASDRLSPGGPDPHHH-----------------------------------------------------------------------
PtCLE34: YKLASWKSGTK-----------------------FKDTIHKAPSGPSPIGNRHRSSIHV------------------------------------------------------------------
PtCLE9 : PSSTEKQQRLR------------------HSFDTFFSSKRKVPNASDPLHNR----------------------------------------------------------------------
PtCLE49: NAKSRMEFHGR------------------RMSISESATDRLSPEGPNHEHHSHPPGNP------------------------------------------------------------------
PtCLE1 : ARASAQDIHQS--------YKITRMGKGASSLVTDEESERRVPTGPDPLHHNNNPTTRP-------------------------------------------------------------------
```
