# Supplementary material for: Identification and characterization of the Populus trichocarpa CLE family
Source: BMC Genomics. 2016 Mar 2;17:174. doi: 10.1186/s12864-016-2504-x (PMC4776436; doi:10.1186/s12864-016-2504-x)
Supplement: Additional file 3: — The multiple sequence alignment of the CLE motifs derived from PtCLE proteins. The conserved residues are shaded in grey. (PDF 28 kb) [file 12864_2016_2504_MOESM3_ESM.pdf]

```
PtCLE4 : LRAAPSGPDPLHH
PtCLE25: LRAVPSGPDPLHH
PtCLE17: SRAVPSGPDPLNN
PtCLE1 : EREVPTGPDPLHH
PtCLE7 : FRLSPGGPDPRHH
PtCLE36: FRLSPGGPDPRHH
PtCLE37: DRVSPGGPDPHHH
PtCLE48: DRLSPGGPDPHHH
PtCLE8 : DRLSPGGPDPQHH
PtCLE16: KRVSPGGPDAKHH
PtCLE35: KRVSPGGPDAQHH
PtCLE43: KRLSPGGPDPKHH
PtCLE50: KRISPGGPDPKHH
PtCLE49: DRLSPEGPNHEHH
PtCLE21: KRLVPTGPNPLHH
PtCLE31: KRLVPTGPNPLHH
PtCLE39: KRLVPTGPNPLHH
PtCLE19: KRRAPRGSDPIHN
PtCLE33: KRRVRRGSDPIHN
PtCLE28: KRRVPNGPDPIHN
PtCLE47: KRRVPNGPDPIHN
PtCLE24: KRKVPNGPDPIHN
PtCLE9 : KRKVPNASDPLHN
PtCLE22: KRRVPSCPDPLHN
PtCLE30: KRRVPSCPDPLHN
PtCLE42: NRVVPSCPDPIHN
PtCLE20: KRLVPSGPNPLHN
PtCLE32: KRLVPSGPNPLHN
PtCLE41: RRLVPSGPNPLHN
PtCLE46: RRLVPCGPNPLHN
PtCLE23: KRIIHTGPNPLHN
PtCLE29: KRTIHTGPNPLHN
PtCLE6 : KRKIFTGPNPLHN
PtCLE18: KRKVYTGPNPLHN
PtCLE27: KRKVYTGPNPLHN
PtCLE44: KRKVPTGSNPLHN
PtCLE11: RRKIPAGPNPLHN
PtCLE10: KRRVPAGPNPLHN
PtCLE40: HKAVPGGPNPLHN
PtCLE45: HKLVPGGPNPLHN
PtCLE13: YRAVPGGPNPLHN
PtCLE2 : AHEVPSGPNPESN
PtCLE15: FHEVPSGPNPESN
PtCLE3 : AHEVPSGPNPISN
PtCLE12: AHEVPSGPNPISN
PtCLE14: AHEVPSGPNPISN
PtCLE38: AHEVPSGPNPISN
PtCLE5 : IHKSPSGPNPVGN
PtCLE26: IHKSSSGPNPVGN
PtCLE34: IHKAPSGPSPIGN
```
