# Supplementary material for: Identification and characterization of the Populus trichocarpa CLE family
Source: BMC Genomics. 2016 Mar 2;17:174. doi: 10.1186/s12864-016-2504-x (PMC4776436; doi:10.1186/s12864-016-2504-x)
Supplement: Additional file 4: — The multiple sequence alignment of all AtCLE and PtCLE proteins using their full-length proteins. The CLE motifs were boxed in red. The conserved residues are shaded in grey. (PDF 46 kb) [file 12864_2016_2504_MOESM4_ESM.pdf]

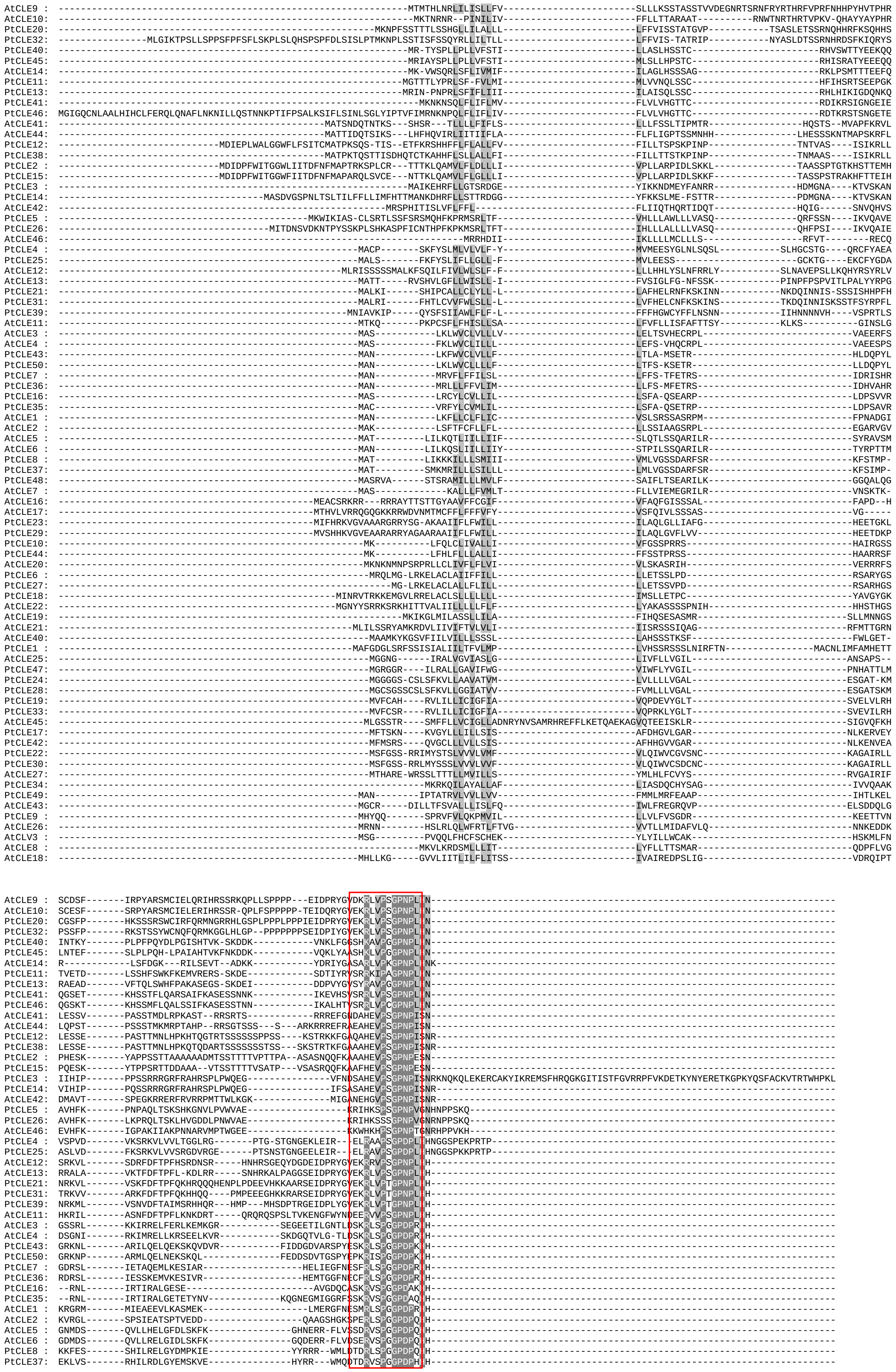

```
PtCLE48:  NANNS-------RHLLLELGFDLSKLE---------------HYRRLSTLSVASDRLSPGGPDPHHH------------------------------------------------------------------------
AtCLE7 :  -DGES-------NDLLKRLGYNVSELK---------------RIGRELSVQNEVDRFSPGGPDPQHHSYPLSSKPRI--------------------------------------------------------------
AtCLE16:  YPSLP-------RKAGHFHEMASFQAPKATVSFTGQ----RREEENRDEVYKDDKRLVHTGPNPLHN------------------------------------------------------------------------
AtCLE17:  YSRL------------HLVASPPPPPPRKALRYSTAP---FRGPLSRDDIYGDDKRVVHTGPNPLHN------------------------------------------------------------------------
PtCLE23:  VKSLP-------RKARFFET-RFHAPPSQDQPLDID-------KGDPDTVYEDDKRIIHTGPNPLHN------------------------------------------------------------------------
PtCLE29:  VKSLP-------RKARVFETGSVHASPNQDQPVNID-------GGDPDAVYEDDKRTIHTGPNPLHN------------------------------------------------------------------------
PtCLE10:  SAPS---------TSQQVFRS----------PFSPS------PFAQRAEEFASQKRRVPAGPNPLHNKR----------------------------------------------------------------------
PtCLE44:  SAPS---------TSQQVFRS----------PFRAS------PFAERAKEFESQKRKVPTGSNPLHNKR----------------------------------------------------------------------
AtCLE20:  SKPS---------GENREFLP----------SQPTF------PVVD-AGEILPDKRKVKTGSNPLHNKR----------------------------------------------------------------------
PtCLE6 :  SKNT---------GSTSQLMG-PVKSHG--GGLRGD------KDEGGDATLGDEKRKIFTGPNPLHNR-----------------------------------------------------------------------
PtCLE27:  FKNT---------GSTAQLTG-PVKSHG--GGLRGD------RDEEGDATLGDEKRKVYTGPNPLHNR-----------------------------------------------------------------------
PtCLE18:  FSSVK-------GGSSSELRNNPAMSNSV-GGLKRN------ANKDGNEIFGADKRKVYTGPNPLHNR-----------------------------------------------------------------------
AtCLE22:  LKKS--------GNLDPKLHDLDSNAASSRGSKYTN------YEGGGEDVFEDGKRRVFTGPNPLHNR-----------------------------------------------------------------------
AtCLE19:  --------------YEEEEQVLKYDS--MGTIANSS--------------ALDSKRVIPTGPNPLHNR-----------------------------------------------------------------------
AtCLE21:  RNLSVARS----LYYKNHHKVVITEMSNFNKVRRRSS---RFRRKTDGDEEEEEKRSIPTGPNPLHNK-----------------------------------------------------------------------
AtCLE40:  ---------------QDTKAMKKEKK--IDGGT----------------ANEVEERQVPTGSDPLHHKHIPFT-P----------------------------------------------------------------
PtCLE1 :  VAAGNRAR----ASAQDIHQSYKITR--MGKGASSLV------------TDEESEREVPTGPDPLHHNNNPTTRP----------------------------------------------------------------
AtCLE25:  ----V-------PSSENV--KTLR------FSGK-----------DVNLFHVS-KRKVPNGPDPIHNRKAETSRRPPRV------------------------------------------------------------
PtCLE47:  ARIRV-------PAAGTF--QHLK------LSGRESH---LIRH-DMDLNYVS-KRRVPNGPDPIHNRKTVQSRQPPGQS-----------------------------------------------------------
PtCLE24:  TERTQ-------TVLDSIAQDDLRRRHE-ELIGREKL---VYNP-ELDLNFVMNKRKVPNGPDPIHNRRAGNSRRPPGRA-----------------------------------------------------------
PtCLE28:  TTSRL-------NSVQAT-QNDLKDDHEKDVIGREKL---VYNS-ELDLNYMMSKRRVPNGPDPIHNRRAGNSKRPPGRA-----------------------------------------------------------
PtCLE19:  NQKAQ-------GTAPQS-QRVLKDVDMQGMDTKKS----AHAS-KTFDRSQSNKRRAPRGSDPIHNRERDVYRKAIRENVHRASIISVPQVEIIINRQ----------------------------------------
PtCLE33:  DRKAH-------GTLPHS-QRSLKDVDMQGMDTKKS----AQAN-KTFDPSQTSKRRVRRGSDPIHNRS----------------------------------------------------------------------
AtCLE45:  TLEDQ-------EMLNKN-RRVLEEVNKDKIKAEETQ---ERKN-KTEDSFKSSKRRVRRGSDPIHNKAQPFS------------------------------------------------------------------
PtCLE17:  TKKT-------------------EGS----VNIQDA----------------TVSRAVPSGPDPLNNR-----------------------------------------------------------------------
PtCLE42:  KEKTQ-------NEK--------EGADSEDINGYVA----------------NVNRVVPSCPDPIHNR-----------------------------------------------------------------------
PtCLE22:  QENDMAKFKESGNNIPANNYKSKEEYFRKYFNERGNT---SHGFNKTEKGFEESKRRVPSCPDPLHN------------------------------------------------------------------------
PtCLE30:  QENGMEKFKES-SDITKDNYKSKEKHFRKYFNERANT---SYGFNKTEKGFEENKRRVPSCPDPLHN------------------------------------------------------------------------
AtCLE27:  PET------------PASGKRQEEDLMKKYFG-AGKF---PPVDSFVGKGISESKRIVPSCPDPLHN------------------------------------------------------------------------
PtCLE34:  SVDTR------LKNAQPILRSTRYKLAS----------------WKSGTKFKDTIHKAPSGPSPIGNRHRSSIHV----------------------------------------------------------------
PtCLE49:  DKRLL--------LSKVLNAKSRMEFHG----------------RRMSISESATDRLSPEGPNHEHHSHPPGNP-----------------------------------------------------------------
AtCLE43:  KDRNT-----LMTSKNKNKNEDVQRLFQRYFKGRSFG------LNNTNSRFEDSNRRIPSSPDRLHN------------------------------------------------------------------------
PtCLE9 :  VHGLQ-------NQQPPSSTEKQQRLR------------------HSFDTFFSSKRKVPNASDPLHNR-----------------------------------------------------------------------
AtCLE26:  TKEIT--------TAVNMNNSDAKEIQQELEDGSR----------NDDLSYVASKRKVPRGPDPIHNRFLLLSRFILSLLTNPYPYLHICVLDVSV-------------------------------------------
AtCLV3 :  WQMMM-------MKMESEWVGANGEAEK---------------AKTKGLGLHEELRTVPSGPDPLHHHVNPPRQPRNNFQLP---------------------------------------------------------
AtCLE8 :  VEKDV-------VPAGTDLKQNKAKP--------------------HLPNLFRTMRRVPTGPNPLHHISPPQPGSLNYARN----------------------------------------------------------
AtCLE18:  GPDPLHN-----PPQPSPKHHHWIGVEENNIDRSWN-------YVDYESHHAHSPIHNSPEPAPLYRHLIGV-------------------------------------------------------------------
```
