# Supplementary material for: Identification and characterization of the Populus trichocarpa CLE family
Source: BMC Genomics. 2016 Mar 2;17:174. doi: 10.1186/s12864-016-2504-x (PMC4776436; doi:10.1186/s12864-016-2504-x)
Supplement: Additional file 5: — The multiple sequence alignment of all AtCLE and PtCLE proteins using their CLE motifs. The conserved residues are shaded in grey. (PDF 14 kb) [file 12864_2016_2504_MOESM5_ESM.pdf]

```
PtCLE4 :  LRAAPSGPDPLHH
PtCLE25:  LRAVPSGPDPLHH
AtCLV3 :  LRTVPSGPDPLHH
PtCLE17:  SRAVPSGPDPLNN
AtCLE40:  ERQVPTGSDPLHH
PtCLE1 :  EREVPTGPDPLHH
AtCLE18:  DRQIPTGPDPLHN
AtCLE25:  KRKVPNGPDPIHN
PtCLE24:  KRKVPNGPDPIHN
PtCLE28:  KRRVPNGPDPIHN
PtCLE47:  KRRVPNGPDPIHN
AtCLE45:  KRRVRRGSDPIHN
PtCLE33:  KRRVRRGSDPIHN
PtCLE19:  KRRAPRGSDPIHN
AtCLE26:  KRKVPRGPDPIHN
PtCLE9 :  KRKVPNASDPLHN
PtCLE22:  KRRVPSCPDPLHN
PtCLE30:  KRRVPSCPDPLHN
AtCLE27:  KRIVPSCPDPLHN
AtCLE43:  NRRIPSSPDRLHN
PtCLE42:  NRVVPSCPDPIHN
PtCLE7 :  FRLSPGGPDPRHH
PtCLE36:  FRLSPGGPDPRHH
AtCLE1 :  MRLSPGGPDPRHH
AtCLE3 :  KRLSPGGPDPRHH
AtCLE4 :  KRLSPGGPDPRHH
AtCLE2 :  ERLSPGGPDPQHH
AtCLE6 :  ERVSPGGPDPQHH
AtCLE5 :  DRVSPGGPDPQHH
AtCLE7 :  DRFSPGGPDPQHH
PtCLE8 :  DRLSPGGPDPQHH
PtCLE37:  DRVSPGGPDPHHH
PtCLE48:  DRLSPGGPDPHHH
PtCLE16:  KRVSPGGPDAKHH
PtCLE35:  KRVSPGGPDAQHH
PtCLE43:  KRLSPGGPDPKHH
PtCLE50:  KRISPGGPDPKHH
PtCLE49:  DRLSPEGPNHEHH
AtCLE8 :  MRRVPTGPNPLHH
AtCLE12:  KRRVPSGPNPLHH
AtCLE11:  ERVVPSGPNPLHH
PtCLE21:  KRLVPTGPNPLHH
PtCLE31:  KRLVPTGPNPLHH
PtCLE39:  KRLVPTGPNPLHH
AtCLE13:  KRLVPSGPNPLHH
PtCLE20:  KRLVPSGPNPLHN
PtCLE32:  KRLVPSGPNPLHN
AtCLE10:  KRLVPSGPNPLHN
AtCLE9 :  KRLVPSGPNPLHN
PtCLE41:  RRLVPSGPNPLHN
PtCLE46:  RRLVPCGPNPLHN
AtCLE14:  ARLVPKGPNPLHN
AtCLE16:  KRLVHTGPNPLHN
AtCLE17:  KRVVHTGPNPLHN
AtCLE22:  KRRVFTGPNPLHN
AtCLE20:  KRKVKTGSNPLHN
PtCLE44:  KRKVPTGSNPLHN
PtCLE18:  KRKVYTGPNPLHN
PtCLE27:  KRKVYTGPNPLHN
AtCLE19:  KRVIPTGPNPLHN
AtCLE21:  KRSIPTGPNPLHN
PtCLE23:  KRIIHTGPNPLHN
PtCLE29:  KRTIHTGPNPLHN
PtCLE6 :  KRKIFTGPNPLHN
PtCLE11:  RRKIPAGPNPLHN
PtCLE10:  KRRVPAGPNPLHN
PtCLE40:  HKAVPGGPNPLHN
PtCLE45:  HKLVPGGPNPLHN
PtCLE13:  YRAVPGGPNPLHN
AtCLE41:  AHEVPSGPNPISN
AtCLE44:  AHEVPSGPNPISN
PtCLE3 :  AHEVPSGPNPISN
PtCLE12:  AHEVPSGPNPISN
PtCLE14:  AHEVPSGPNPISN
PtCLE38:  AHEVPSGPNPISN
PtCLE2 :  AHEVPSGPNPESN
PtCLE15:  FHEVPSGPNPESN
AtCLE42:  EHGVPSGPNPISN
PtCLE5 :  IHKSPSGPNPVGN
PtCLE26:  IHKSSSGPNPVGN
PtCLE34:  IHKAPSGPSPIGN
AtCLE46:  WHKHPSGPNPTGN
```
