# Supplementary material for: Identification and characterization of the Populus trichocarpa CLE family
Source: BMC Genomics. 2016 Mar 2;17:174. doi: 10.1186/s12864-016-2504-x (PMC4776436; doi:10.1186/s12864-016-2504-x)

```
          1                18
PtCLE7 : GFNESFRLSPGGPDPRHH
PtCLE36: GFNECFRLSPGGPDPRHH
PtCLE16: DQCASKRVSPGGPDAKHH
PtCLE35: GRFSSKRVSPGGPDAQHH
PtCLE43: SPYESKRLSPGGPDPKHH
PtCLE50: SPYEPKRISPGGPDPKHH
PtCLE8 : WMLDTDRLSPGGPDPQHH
PtCLE37: WMQDTDRVSPGGPDPHHH
PtCLE48: LSVASDRLSPGGPDPHHH
PtCLE49: SESATDRLSPEGPNHEHH
PtCLE1 : TDEESEREVPTGPDPLHH
PtCLE4 : LEIRELRAAPSGPDPLHH
PtCLE25: LEIRELRAVPSGPDPLHH
PtCLE10: EFASQKRRVPAGPNPLHN
PtCLE44: EFESQKRKVPTGSNPLHN
PtCLE9 : TFFSSKRKVPNASDPLHN
PtCLE19: RSQSNKRRAPRGSDPIHN
PtCLE33: PSQTSKRRVRRGSDPIHN
PtCLE24: NFVMNKRKVPNGPDPIHN
PtCLE28: NYMMSKRRVPNGPDPIHN
PtCLE47: LNYVSKRRVPNGPDPIHN
PtCLE22: GFEESKRRVPSCPDPLHN
PtCLE30: GFEENKRRVPSCPDPLHN
PtCLE17: QDATVSRAVPSGPDPLNN
PtCLE42: YVANVNRVVPSCPDPIHN
PtCLE6 : TLGDEKRKIFTGPNPLHN
PtCLE27: TLGDEKRKVYTGPNPLHN
PtCLE18: IFGADKRKVYTGPNPLHN
PtCLE23: VYEDDKRIIHTGPNPLHN
PtCLE29: VYEDDKRTIHTGPNPLHN
PtCLE20: RYGVEKRLVPSGPNPLHN
PtCLE32: IYGVEKRLVPSGPNPLHN
PtCLE21: RYGVEKRLVPTGPNPLHH
PtCLE31: RYGVEKRLVPTGPNPLHH
PtCLE39: LYGVEKRLVPTGPNPLHH
PtCLE41: VHSVSRRLVPSGPNPLHN
PtCLE46: LHTVSRRLVPCGPNPLHN
PtCLE11: IYRVSRRKIPAGPNPLHN
PtCLE40: LFGGSHKAVPGGPNPLHN
PtCLE45: LYAASHKLVPGGPNPLHN
PtCLE13: VYGVSYRAVPGGPNPLHN
PtCLE2 : QFKAAAHEVPSGPNPESN
PtCLE15: QFKAAFHEVPSGPNPESN
PtCLE12: KFGAQAHEVPSGPNPISN
PtCLE38: KFGAAAHEVPSGPNPISN
PtCLE14: IFSASAHEVPSGPNPISN
PtCLE3 : VFNDSAHEVPSGPNPISN
PtCLE5 : VAEKRIHKSPSGPNPVGN
PtCLE26: VAEKRIHKSSSGPNPVGN
PtCLE34: KFKDTIHKAPSGPSPIGN
```

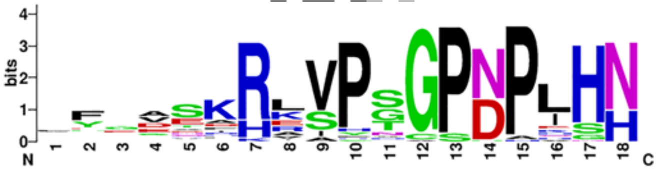

Supplement: Additional file 6: — The multiple sequence alignment of all PtCLE proteins using their CLE motifs and five N-terminal residues flanking the CLE motifs (18-AA in length). The conserved residues are shaded in grey. Weblogo plot was used for graphical representation of the multiple sequence alignment of the 18-AA fragments. (PDF 72 kb) [file 12864_2016_2504_MOESM6_ESM.pdf]
