# Supplementary material for: Identification and characterization of the Populus trichocarpa CLE family
Source: BMC Genomics. 2016 Mar 2;17:174. doi: 10.1186/s12864-016-2504-x (PMC4776436; doi:10.1186/s12864-016-2504-x)

```
          1                  18
AtCLE25:  LFHVSKRKVPNGPDPIHN
PtCLE47:  LNYVSKRRVPNGPDPIHN
PtCLE24:  NFVMNKRKVPNGPDPIHN
PtCLE28:  NYMMSKRRVPNGPDPIHN
AtCLE26:  SYVASKRKVPRGPDPIHN
AtCLE45:  SFKSSKRRVRRGSDPIHN
PtCLE33:  PSQTSKRRVRRGSDPIHN
PtCLE19:  RSQSNKRRAPRGSDPIHN
PtCLE9 :  TFFSSKRKVPNASDPLHN
PtCLE22:  GFEESKRRVPSCPDPLHN
PtCLE30:  GFEENKRRVPSCPDPLHN
AtCLE27:  GISESKRIVPSCPDPLHN
AtCLE43:  RFEDSNRRIPSSPDRLHN
PtCLE4 :  LEIRELRAAPSGPDPLHH
PtCLE25:  LEIRELRAVPSGPDPLHH
AtCLV3 :  GLHEELRTVPSGPDPLHH
AtCLE40:  ANEVEERQVPTGSDPLHH
PtCLE1 :  TDEESEREVPTGPDPLHH
AtCLE8 :  NLFRTMRRVPTGPNPLHH
PtCLE7 :  GFNESFRLSPGGPDPRHH
PtCLE36:  GFNECFRLSPGGPDPRHH
AtCLE1 :  RFNESMRLSPGGPDPRHH
AtCLE7 :  VQNEVDRFSPGGPDPQHH
PtCLE43:  SPYESKRLSPGGPDPKHH
PtCLE50:  SPYEPKRISPGGPDPKHH
AtCLE5 :  FLVSSDRVSPGGPDPQHH
AtCLE6 :  FLVDSERVSPGGPDPQHH
AtCLE2 :  HGKSPERLSPGGPDPQHH
PtCLE8 :  WMLDTDRLSPGGPDPQHH
PtCLE37:  WMQDTDRVSPGGPDPHHH
PtCLE48:  LSVASDRLSPGGPDPHHH
PtCLE16:  DQCASKRVSPGGPDAKHH
PtCLE35:  GRFSSKRVSPGGPDAQHH
AtCLE3 :  NTLDSKRLSPGGPDPRHH
AtCLE4 :  GTLDSKRLSPGGPDPRHH
PtCLE49:  SESATDRLSPEGPNHEHH
AtCLE10:  RYGVEKRLVPSGPNPLHN
PtCLE20:  RYGVEKRLVPSGPNPLHN
AtCLE9 :  RYGVDKRLVPSGPNPLHN
PtCLE32:  IYGVEKRLVPSGPNPLHN
AtCLE12:  RYGVEKRRVPSGPNPLHH
AtCLE13:  RYGVEKRLVPSGPNPLHH
PtCLE21:  RYGVEKRLVPTGPNPLHH
PtCLE31:  RYGVEKRLVPTGPNPLHH
PtCLE39:  LYGVEKRLVPTGPNPLHH
AtCLE11:  WYNDEERVVPSGPNPLHH
PtCLE40:  LFGGSHKAVPGGPNPLHN
PtCLE45:  LYAASHKLVPGGPNPLHN
PtCLE13:  VYGVSYRAVPGGPNPLHN
AtCLE14:  IYGASARLVPKGPNPLHN
PtCLE41:  VHSVSRRLVPSGPNPLHN
PtCLE46:  LHTVSRRLVPCGPNPLHN
PtCLE11:  IYRVSRRKIPAGPNPLHN
AtCLE19:  SALDSKRVIPTGPNPLHN
AtCLE21:  EEEEEKRSIPTGPNPLHN
AtCLE18:  SLIGVDRQIPTGPDPLHN
PtCLE23:  VYEDDKRIIHTGPNPLHN
PtCLE29:  VYEDDKRTIHTGPNPLHN
AtCLE16:  VYKDDKRLVHTGPNPLHN
AtCLE17:  IYGDDKRVVHTGPNPLHN
AtCLE22:  VFEDGKRRVFTGPNPLHN
PtCLE6 :  TLGDEKRKIFTGPNPLHN
PtCLE27:  TLGDEKRKVYTGPNPLHN
PtCLE18:  IFGADKRKVYTGPNPLHN
AtCLE20:  EILPDKRKVKTGSNPLHN
PtCLE44:  EFESQKRKVPTGSNPLHN
PtCLE10:  EFASQKRRVPAGPNPLHN
PtCLE17:  QDATVSRAVPSGPDPLNN
PtCLE42:  YVANVNRVVPSCPDPIHN
AtCLE41:  EFGNDAHEVPSGPNPISN
AtCLE44:  EFRAEAHEVPSGPNPISN
PtCLE12:  KFGAQAHEVPSGPNPISN
PtCLE38:  KFGAAAHEVPSGPNPISN
PtCLE14:  IFSASAHEVPSGPNPISN
PtCLE3 :  VFNDSAHEVPSGPNPISN
PtCLE2 :  QFKAAAHEVPSGPNPESN
PtCLE15:  QFKAAFHEVPSGPNPESN
AtCLE42:  MIGANEHGVPSGPNPISN
PtCLE5 :  VAEKRIHKSPSGPNPVGN
PtCLE26:  VAEKRIHKSSSGPNPVGN
AtCLE46:  GEEKKWHKHPSGPNPTGN
PtCLE34:  KFKDTIHKAPSGPSPIGN
```

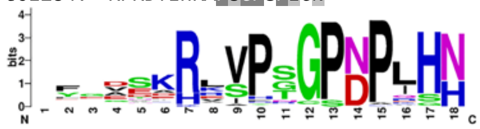

Supplement: Additional file 7: — The multiple sequence alignment of all AtCLE and PtCLE proteins using their CLE motifs and five N-terminal residues flanking the CLE motifs (18-AA in length). The conserved residues are shaded in grey. Weblogo plot was used for graphical representation of the multiple sequence alignment of the 18-AA fragments. (PDF 58 kb) [file 12864_2016_2504_MOESM7_ESM.pdf]
