# Supplementary figures and images for: Identification and characterization of the Populus trichocarpa CLE family
Source: BMC Genomics. 2016 Mar 2;17:174. doi: 10.1186/s12864-016-2504-x (PMC4776436; doi:10.1186/s12864-016-2504-x)

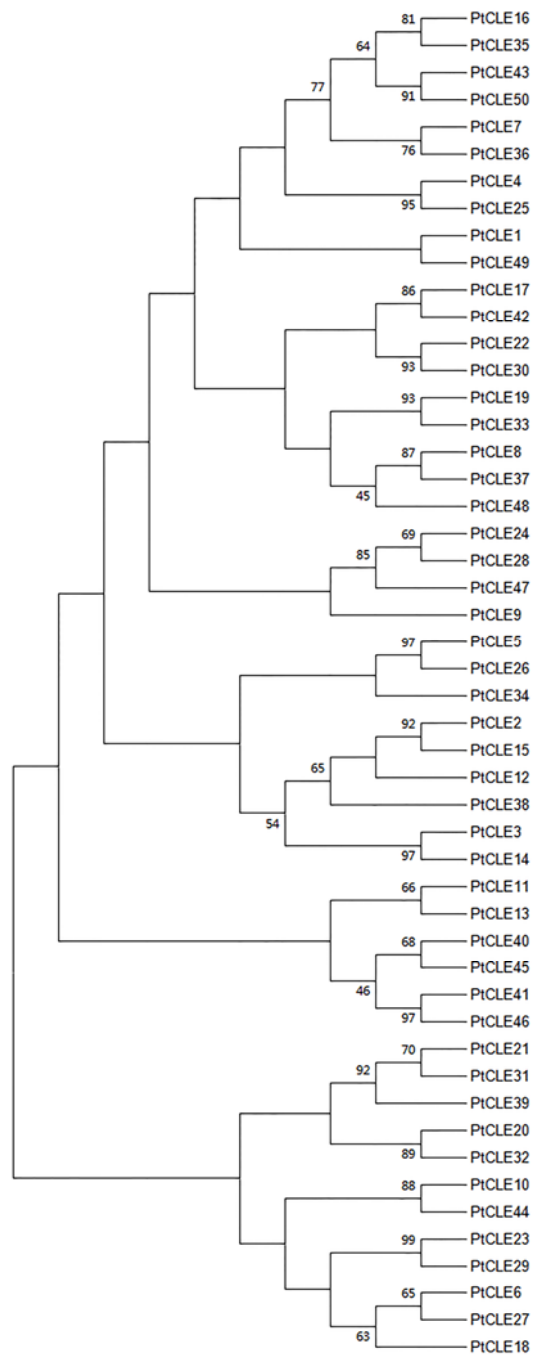

Supplement: Additional file 8: — Phylogenetic analysis of PtCLE proteins by the Neighbor-joining method with 1000 bootstrap iterations. The tree was constructed using full-length PtCLE proteins. The percentage of trees in which the associated clades clustered together is shown (>40 %). (PDF 107 kb) [file 12864_2016_2504_MOESM8_ESM.pdf]

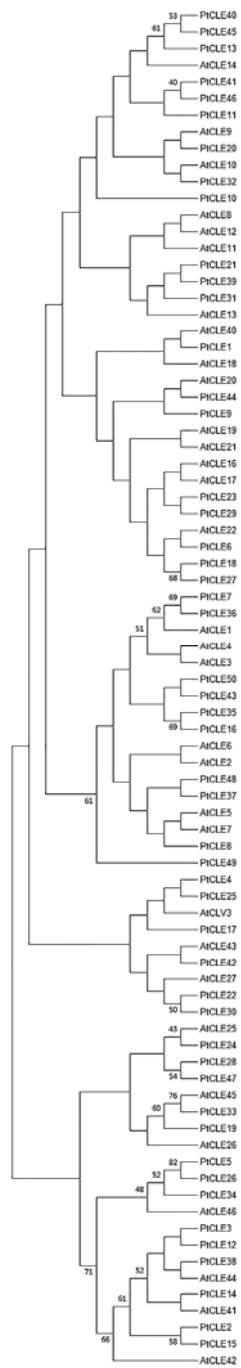

Supplement: Additional file 10: — Phylogenetic analysis of AtCLE and PtCLE proteins by the Neighbor-joining method with 1000 bootstrap iterations. The tree was constructed using the conserved CLE motifs. The percentage of trees in which the associated clades clustered together is shown (>40 %). (PDF 70 kb) [file 12864_2016_2504_MOESM10_ESM.pdf]

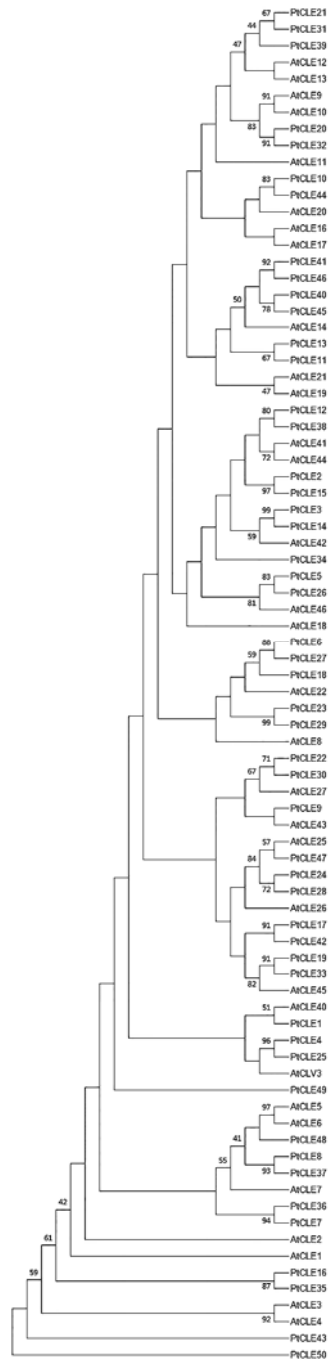

Supplement: Additional file 11: — Phylogenetic analysis of AtCLE and PtCLE proteins by the Neighbor-joining method with 1000 bootstrap iterations. The tree was constructed using full-length proteins. The percentage of trees in which the associated clades clustered together is shown (>40 %). (PDF 39 kb) [file 12864_2016_2504_MOESM11_ESM.pdf]

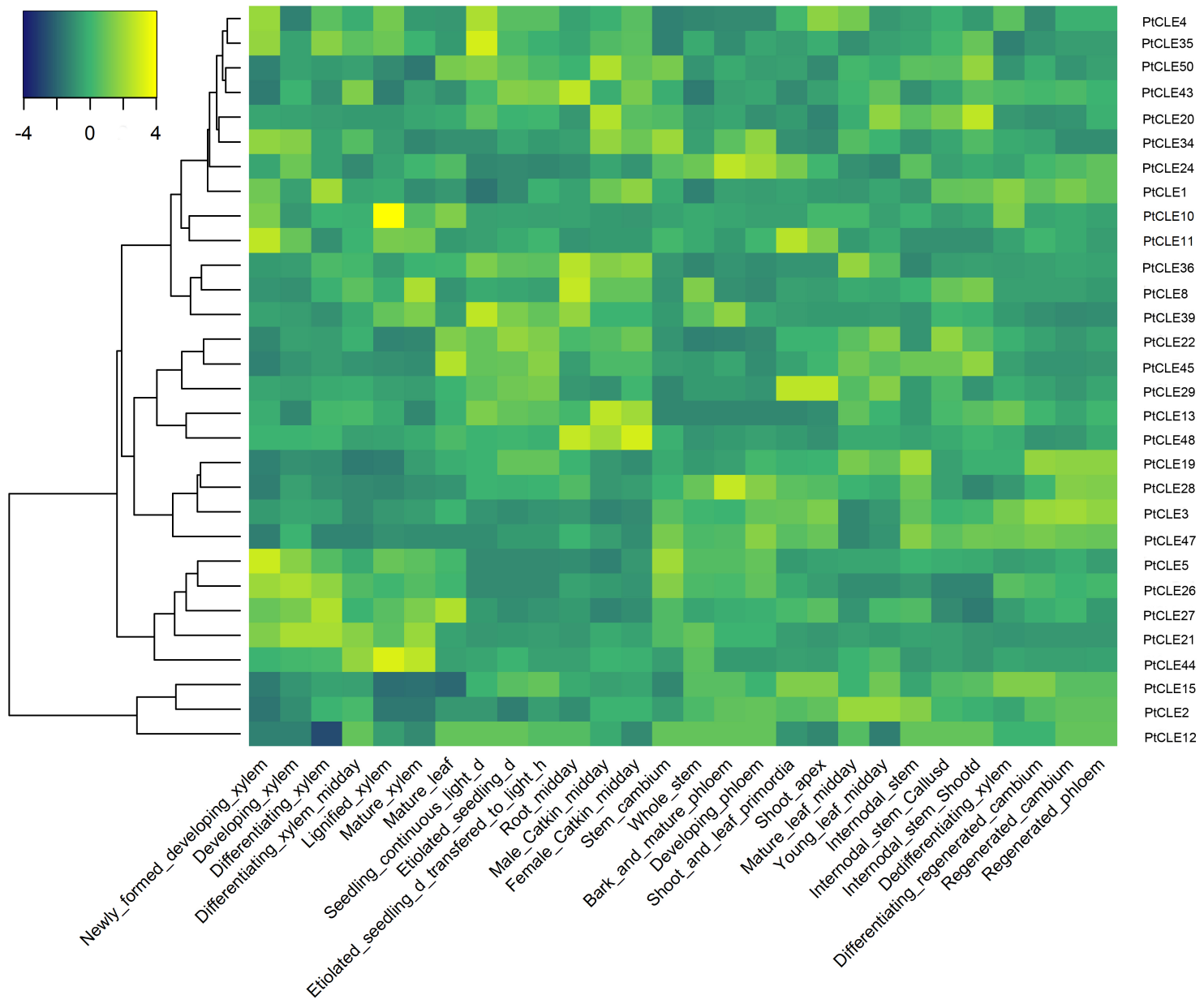

Supplement: Additional file 12: — Transctiptional profiling of PtCLE genes in various organs and tissues using the available microarray data. The microarray data were downloaded from GEO and normalized for analysis. Color scale represents log2 expression values. (PDF 2632 kb) [file 12864_2016_2504_MOESM12_ESM.pdf]

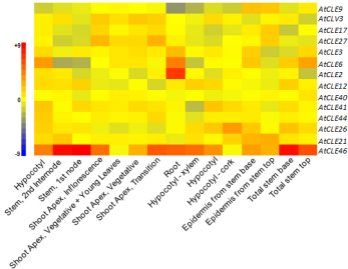

Supplement: Additional file 14: — The expression pattern of AtCLE genes in shoot- and vascular-related tissues. Gene expression is displayed as normalized log2-transformed values. (PDF 148 kb) [file 12864_2016_2504_MOESM14_ESM.pdf]

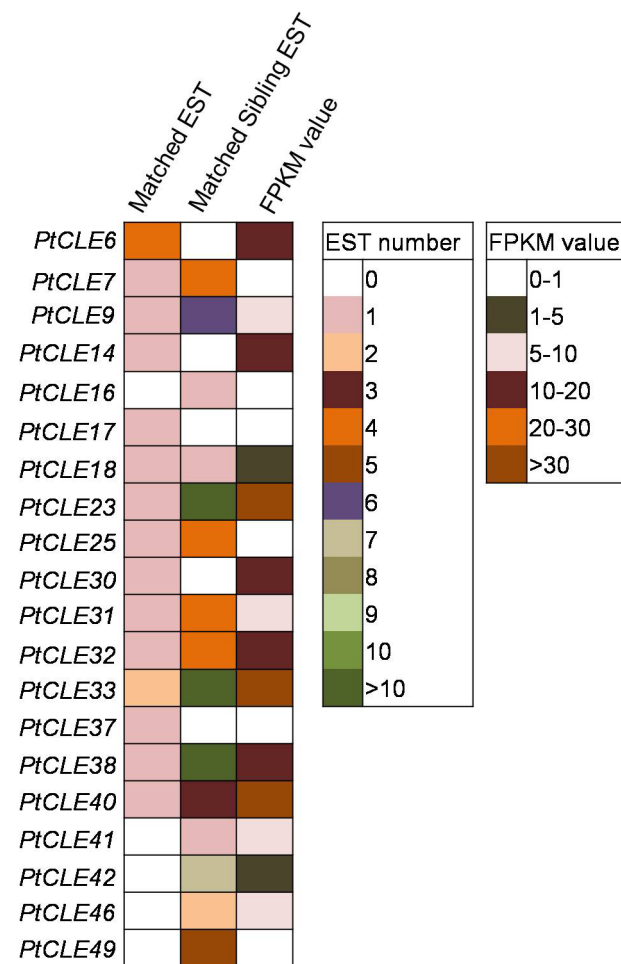

Supplement: Additional file 15: — The EST sequences and RNA-seq data for PtCLE genes which are not presented in the microarray. The expression level for RNA-seq data was presented as numbers of fragments per kilobase of exon in a gene per million fragments mapped (FPKM). (PDF 237 kb) [file 12864_2016_2504_MOESM15_ESM.pdf]
