# Supplementary material for: Identification and characterization of the Populus trichocarpa CLE family
Source: BMC Genomics. 2016 Mar 2;17:174. doi: 10.1186/s12864-016-2504-x (PMC4776436; doi:10.1186/s12864-016-2504-x)
Supplement: Additional file 9: — A list of AtCLE and PtCLE proteins with identical CLE motifs. (PDF 12 kb) [file 12864_2016_2504_MOESM9_ESM.pdf]

Gene symbol	CLE motif(13aa)
AtCLE2	E**RLSPGGPDPQHH**
PtCLE8	D**RLSPGGPDPQHH**
AtCLE9	RLVPSGPNPLHN
AtCLE10	RLVPSGPNPLHN
PtCLE20	RLVPSGPNPLHN
PtCLE32	RLVPSGPNPLHN
AtCLE25	KRKVPNGPDPIHN
PtCLE24	KRKVPNGPDPIHN
AtCLE41	AHEVPSGPNPISN
AtCLE44	AHEVPSGPNPISN
PtCLE3	AHEVPSGPNPISN
PtCLE12	AHEVPSGPNPISN
PtCLE14	AHEVPSGPNPISN
PtCLE38	AHEVPSGPNPISN
AtCLE45	EHGVPSGPNPISN
PtCLE33	EHGVPSGPNPISN
PtCLE7	FRLSPGGPDPRHH
PtCLE36	FRLSPGGPDPRHH
PtCLE18	KRKVYTGPNPLHN
PtCLE27	KRKVYTGPNPLHN
PtCLE20	KRLVPSGPNPLHN
PtCLE32	KRLVPSGPNPLHN
PtCLE21	KRLVPTGPNPLHH
PtCLE31	KRLVPTGPNPLHH
PtCLE39	KRLVPTGPNPLHH
PtCLE22	KRRVPSCPDPLHN
PtCLE30	KRRVPSCPDPLHN
PtCLE28	KRRVPNGPDPIHN
PtCLE47	KRRVPNGPDPIHN
