# Supplementary material for: Identification and characterization of the Populus trichocarpa CLE family
Source: BMC Genomics. 2016 Mar 2;17:174. doi: 10.1186/s12864-016-2504-x (PMC4776436; doi:10.1186/s12864-016-2504-x)
Supplement: Additional file 13: — A list of microarray datasets used in this study. Note that available microarray data were derived from different Populus species other than P. trichocarpa. (PDF 31 kb) [file 12864_2016_2504_MOESM13_ESM.pdf]

Additional file 13. A list of microarray data sets used in this study

**GEO Accession**	**Species**	**Tissues**
GSE12152	*Populus tremula* × *Populus alba*	Callus induction and shoot formation
GSE13990	*Populus balsamifera*	Developmental tissue series
GSE25309	*Populus tomentosa*	Secondary vascular tissue regeneration
GSE30507	*Populus trichocarpa*	Developing xylem
GSE46312	*Populus tomentosa*	Two year old shoot apex and mature xylem
GSE46946	*Populus tomentosa*	Newly formed developing xylem and lignified xylem
